# Suppression of antibiotic resistance evolution by single-gene deletion

**DOI:** 10.1038/s41598-020-60663-6

**Published:** 2020-03-06

**Authors:** Takaaki Horinouchi, Tomoya Maeda, Hazuki Kotani, Chikara Furusawa

**Affiliations:** 1 Center for Biosystems Dynamics Research, RIKEN, 6-2-3 Furuedai, Suita Osaka, 565-0874 Japan; 2 Universal Biology Institute, The University of Tokyo, 7-3-1 Hongo, Tokyo, 113-0033 Japan

**Keywords:** Evolvability, Evolvability, Evolvability, Genetic interaction, Genetic interaction

## Abstract

Antibiotic treatment generally results in the selection of resistant bacterial strains, and the dynamics of resistance evolution is dependent on complex interactions between cellular components. To better characterize the mechanisms of antibiotic resistance and evaluate its dependence on gene regulatory networks, we performed systematic laboratory evolution of *Escherichia coli* strains with single-gene deletions of 173 transcription factors under three different antibiotics. This resulted in the identification of several genes whose deletion significantly suppressed resistance evolution, including *arcA* and *gutM*. Analysis of double-gene deletion strains suggested that the suppression of resistance evolution caused by *arcA* and *gutM* deletion was not caused by epistatic interactions with mutations known to confer drug resistance. These results provide a methodological basis for combinatorial drug treatments that may help to suppress the emergence of resistant pathogens by inhibiting resistance evolution.

## Introduction

The emergence of virtually untreatable multidrug-resistant bacteria is an increasing public health concern worldwide^1,2^. Bacterial cells inevitably evolve resistance when this offers a selective advantage under clinical doses of antibiotics, resulting in ineffective antibiotic treatment^3^. Although newly developed compounds have been introduced to treat such resistant bacteria^4^, further resistance acquisition to these compounds often occurs^5^. Furthermore, the development of new antibiotics has declined recently^6^, hence there is a need to develop novel methodologies to suppress the emergence of antibiotic-resistant bacteria.

One approach to combat drug resistance is the use of multidrug combinations, either sequentially or simultaneously^7,8^. It has been demonstrated that drug pairs with synergistic interactions can suppress the emergence of resistance by reducing the time window available for resistance evolution^8^. Furthermore, drug pairs with antagonistic interactions have been shown to suppress resistance evolution by limiting the range of drug concentrations in which resistance is selected^9,10^. In addition to these synergistic and antagonistic drug interactions, collateral sensitivity^11^, i.e., the phenomenon where resistance evolution to one drug induces sensitivity to another, is expected to play an important role in the development of therapeutic strategies to control resistance evolution^12^. Cycling doses and simultaneous addition of drug pairs displaying collateral sensitivity have been shown to result in significant suppression of resistance evolution, providing valuable information for the design of future drug regimes^12,13^.

Several studies have identified tight networks of drug-drug interactions and collateral resistance/sensitivity among drugs^14–16^, indicating complex interactions between the mechanisms of drug responses and resistance evolution. For example, several studies have demonstrated that the collateral sensitivity between β-lactam and aminoglycoside observed in *Escherichia coli (E. coli)* is caused by changes in the proton-motive force (PMF) across the cell membrane and respiratory activity^16,17^. Through these studies it has become clear that resistance evolution involves changes in various cellular functions, such as metabolic activity, membrane transport, transcription, and translation. Thus, to design effective mechanisms to suppress resistance evolution, we need to decipher the complex interactions between resistance evolution and these cellular functions. However, in previous studies, the analyses of such interactions have been limited to responses and resistance acquisition to known antibiotics, resulting in a failure to reveal critical molecular mechanisms affecting the dynamics of resistance evolution.

In this study, to systematically investigate mechanisms to suppress antibiotic resistance evolution, we performed laboratory evolution of single-gene deletion strains^18^ of *E. coli* in the presence of three antibiotics with different targeting mechanisms. We used deletion strains of transcription factors (TFs) as the ancestors of the laboratory evolution, as their deletion is expected to perturb a wide range of cellular functions. We screened for TF genes whose deletion significantly suppressed or accelerated antibiotic resistance evolution. Based on the results, we discuss strategies to develop drug combinations that could inhibit antibiotic resistance evolution thereby improving the success of future antibiotic treatments.

## Results

### Laboratory evolution of single-gene deletion strains under antibiotics

**Figure 1:**
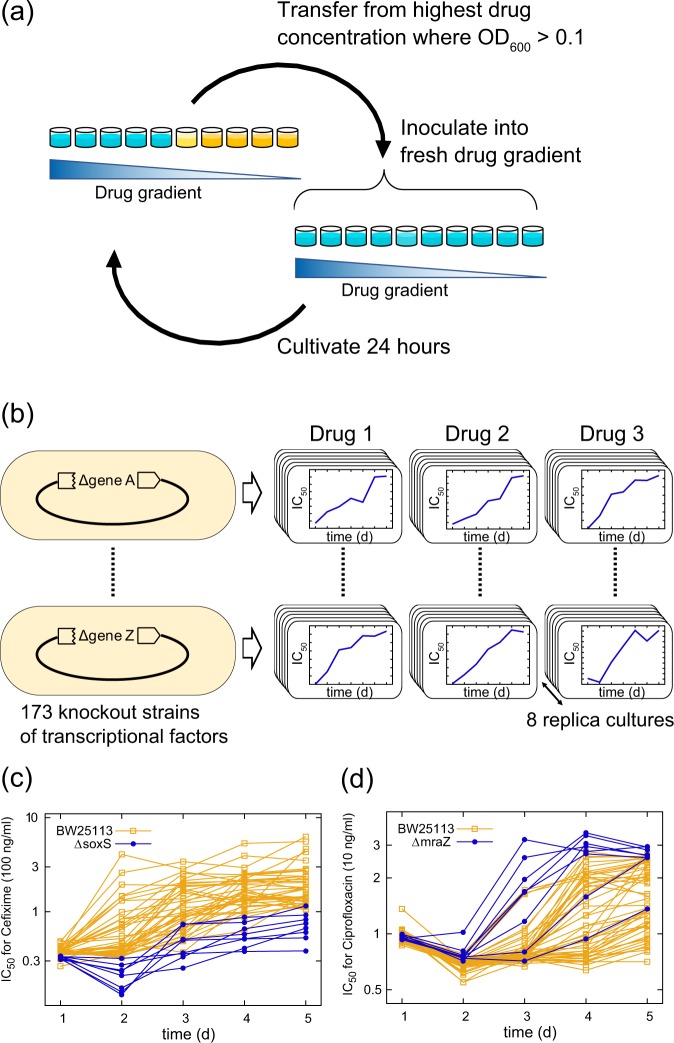
Laboratory evolution using single-gene deletion strains as ancestors. (**a**) The method of laboratory evolution by serial dilution. Every 24 hours, cells from the well with the highest drug concentration that exhibited an OD_620_ > 0.1 were transferred to fresh medium with the drug gradient. (**b**) Design of the experiment. We used 173 deletion strains of transcription factors as the ancestor strains for laboratory evolution in three different antibiotics. (**c,d**) Examples of the time courses of IC_50_ during laboratory evolution. The IC_50_ time courses of (**c**) the Δ*soxS* strain under Cefixime and (**d**) the Δ*mraZ* strain under Ciprofloxacin (blue lines) are presented with those of the wild-type strain BW25113 (without gene deletion - yellow lines). The time courses of 8 and 40 replicates are shown for the deletion strains and BW25113, respectively.

Figure 1 shows a schematic of the experimental design of this study. To investigate the effect of gene deletion on antibiotic resistance evolution, we evolved *E. coli* strains obtained from the Keio single-gene deletion library^18^ in the presence of 3 antibiotics. The drugs cover three major antibiotic targets in *E. coli*: cell wall synthesis (Cefixime; CFIX), DNA replication (Ciprofloxacin; CPFX), and protein synthesis (Chloramphenicol; CP). We selected 173 deletion strains of TFs from the deletion library as ancestors of the laboratory evolution (the list of deleted genes is shown in Table S1).

The laboratory evolution experiments were carried out using 2^0.5^ (CFIX and CPFX) or 2^0.25^ (CP)-fold dilution gradients in 384-well plates. Cells were propagated daily from the well containing the highest drug concentration that exceeded a given threshold of an optical density at 620 nm (OD_620_) (Fig. 1a; see Materials and methods for details). To evaluate the reproducibility of the evolutionary dynamics, 8 independent culture lines were propagated in parallel for each antibiotic/ancestor combination. The wild-type strain BW25113, which is the host strain of the gene deletion library, was used as a control. More than 4,000 independent culture series were maintained (173 deletion strains ×3 drugs ×8 replicates and controls; Fig. 1b). The evolution was performed for 5 passages (CFIX and CPFX) or 8 passages (CP), resulting in significant increases in drug resistance in the wild-type strain BW25113 (without gene deletion). All experiments were performed by an automated culture system that we previously developed for laboratory evolution^19^.

To quantify drug resistance, we calculated 50% inhibition levels (IC_50_) from the OD_620_ measurements in the daily propagation. Figures 1c,d show examples of the time course of IC_50_ during laboratory evolution. The yellow lines represent the resistance evolution of BW25113 (without gene deletion, n = 40), while the blue lines correspond to the resistance evolution of Δ*soxS* under CFIX (Fig. 1c) and Δ*mraZ* under CPFX (Fig. 1d) (n = 8). The deletion of *soxS*, encoding the activator of the superoxide response regulon^20^, suppressed resistance evolution to the cell wall synthesis inhibitor CFIX, whereas the deletion of *mraZ*, encoding a repressor that controls cell division and cell wall synthesis^21^, accelerated resistance evolution to the DNA replication inhibitor CPFX. All plots for the 173 deletion strains are shown in Figs. S1–S3 and the IC_50_ values are presented in Table S2.

### Impact of gene deletion on resistance evolution

**Figure 2:**
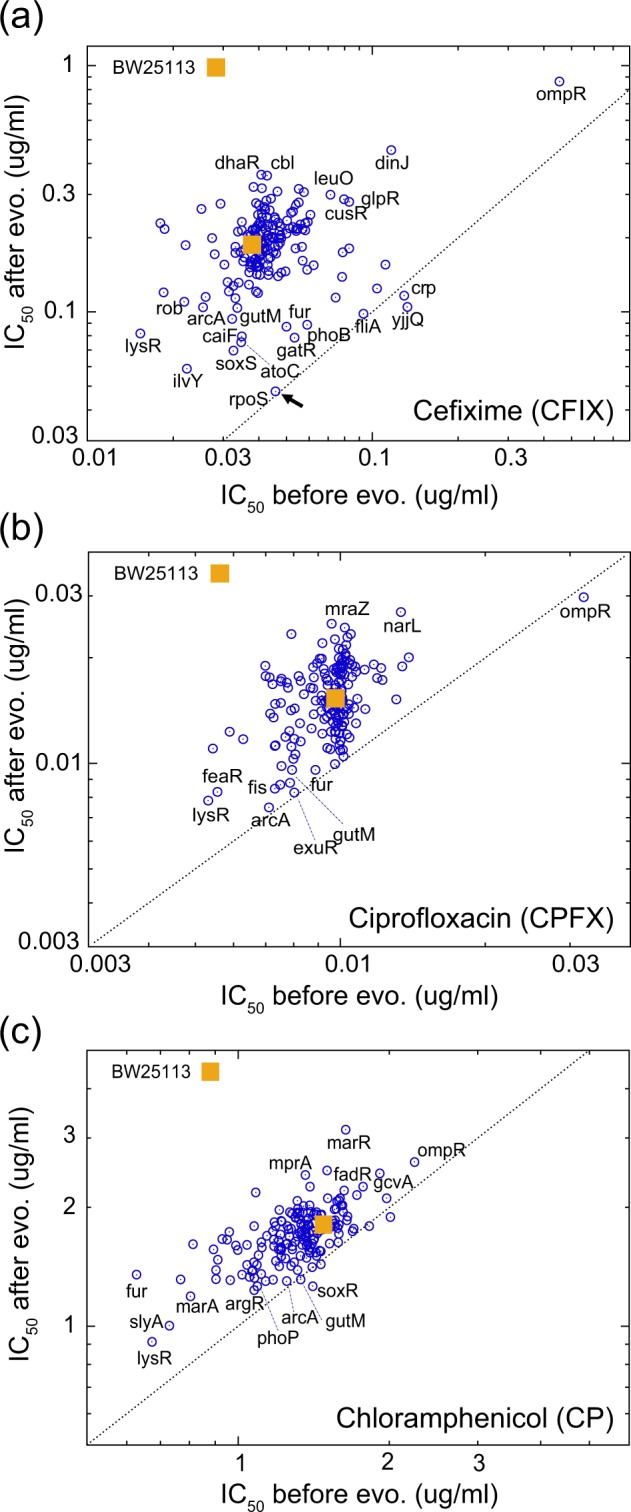
The relationship between IC_50_ values before and after laboratory evolution. Each dot represents the mean IC_50_ of 173 deletion strains over 8 replica culture series for (**a**) CFIX, (**b**) CPFX, and (**c**) CP. The average IC_50_ of the wild-type strain BW25113 (without deletion) for each drug is also presented as the yellow square. Names of some representative genes whose deletion strains show large deviations from the BW25113 control are presented proximal to the corresponding data points. The dotted line shows the function *y* = *x* for reference.

Figure 2 shows the relationship between drug resistance before and after evolution, where each dot represents the mean IC_50_ of the 8 replica culture series for the 173 deletion strains. The data for the wild-type strain BW25113 (without deletion) are also plotted. The *x*-axis and *y*-axis show the IC_50_ values calculated from the dose-response curves on the first day and the last day, respectively. Most data points are located above the dotted reference line (*y* = *x*), indicating an increase in drug resistance during the laboratory evolution.

In Fig. 2, the variance of IC_50_ values on the *x*-axis represents the effect of deleting TFs on the drug resistance. For example, the deletion of *ompR* exhibited a significant increase in IC_50_ to all three drugs. It is known that the deletion of *ompR* causes decreased expression of OmpF outer membrane porins^22^, which leads to resistance to various drugs. In contrast, the deletion of *lysR* causes sensitivity to all three drugs we investigated.

The IC_50_ values on the *y*-axis provide information on how the deletion of TFs affects the resistance evolution. For example, before laboratory evolution, the deletion strain of *rpoS*, encoding sigma factor σ^s^ of RNA polymerase^23^, exhibited a similar IC_50_ to CFIX as the wild-type strain BW25113, and there was little increase of IC_50_ during the laboratory evolution (depicted by an arrow in Fig. 2a). On the other hand, the deletion strain of *rpoS* acquired resistance to CPFX and CP by laboratory evolution (Fig. S4), which may suggest that *rpoS*-mediated regulation plays a more important role in the resistance evolution to CFIX than the other two drugs. The advantage of systematic laboratory evolution is that we can distinguish the effect of gene deletion on drug resistance from its impact on resistance evolution.

One possible explanation for the changes in IC_50_ shown in Fig. 2 is changing growth rate caused by deletion of TFs. To check this possibility, we quantified the specific growth rate and the cell concentration at the stationary phase for the TF deletion strains, and analyzed the relationship to the IC_50_ values after evolution under antibiotics. As shown in Fig. S5, there was no correlation between the change in IC_50_ and the growth rate/final cell concentration in the TF deletion strains. This result suggests that the change of IC_50_ shown in Fig. 2 is not caused by changes in the growth rate. Another possible explanation of the changes in resistance evolution in Fig. 2 is the changing mutation rate by the TF deletions. To check this possibility, we compared the change in mutation rate of 12 TF deletion strains obtained by previous study^24^ and IC_50_ values on the last day, as shown in Fig. S6. The results demonstrated that there was no correlation between the change in the mutation rate and the observed changes in the resistance evolution.

### Genes whose deletion suppresses antibiotic resistance evolution

**Figure 3:**
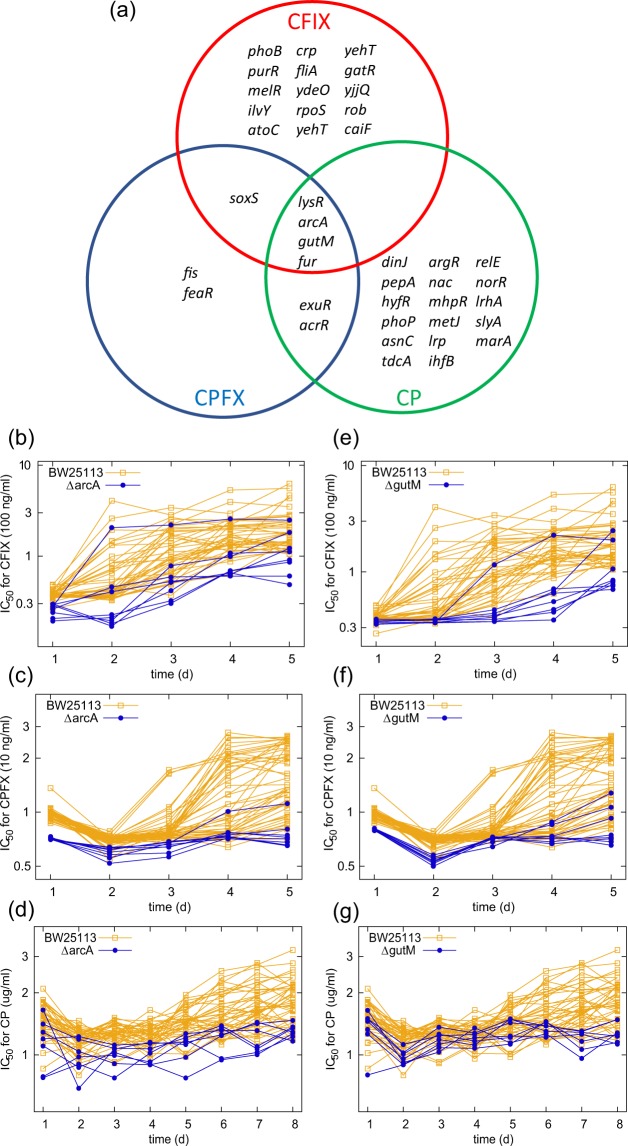
Genes whose deletion significantly suppresses resistance evolution. (**a**) Venn diagram representing genes whose deletion resulted in significantly lower IC_50_ values for the indicated drugs (Cefixime; CFIX, Ciprofloxacin; CPFX, Chloramphenicol; CP) on the last day of laboratory evolution in comparison with the wild-type strain BW25113 (without gene deletion). The genes with FDR (calculated by Benjamini-Hochberg method) <0.1 are presented. (**b–g**) The time courses of IC_50_ for Δ*arcA* (**b–g**) and Δ*gutM* (**e–g**) in the laboratory evolution with the three indicated drugs.

Based on the time series data of IC_50_ during the laboratory evolution, we screened genes whose deletion significantly suppressed resistance evolution. For the screening, we used the Wilcoxon rank sum test between IC_50_ values of a deletion strain and the wild-type BW25113 strain on the last day of laboratory evolution. The Venn diagram in Fig. 3a shows the 42 genes whose deletion strains exhibited significantly lower IC_50_ values than the control strain (false discovery rate (FDR) is less than 0.1). The Venn diagram for the acceleration of resistance evolution, containing a total of 21 genes, is shown in Fig. S7. As can be seen in Fig. 3a, the deletion strains of four genes (*lysR*, *arcA*, *gutM*, *fur*) had significantly lower IC_50_ values for all three drugs. Of these, the Δ*lysR* and Δ*fur* strains also exhibited significantly lower IC_50_ than other strains on the first day, as demonstrated that they can be statistically excluded at outliers (*p* < 0.01; chi-squared test for outliers) in the case of CP resistance. Here, we exclude Δ*lysR* and Δ*fur* from the following analysis, since for these strains, the resistance acquisition during laboratory evolution was difficult to be evaluated by the IC_50_ values on the last day. In this analysis, many deletion strains exhibited significantly different resistance levels on the first day from the wild-type strain (Table S2). However, the absolute differences in IC_50_ values are not always large, and in this study, we focus on the genes whose deletion strains showed similar IC_50_ values on the first day, and different IC_50_ values after evolution, as Δ*arcA* and Δ*gutM s*trains. Figures 3b–g show the time course of IC_50_ during the adaptive evolution of Δ*arcA* and Δ*gutM*, respectively. These deletion strains exhibited similar IC_50_ values as BW25113 and other deletion strains before the adaptive evolution, while the resistance acquisitions were significantly suppressed.

### Analysis of epistatic interactions

As shown in Fig. 3b–g, the deletion of *arcA* and *gutM* suppressed resistance evolution to the three drugs CFIX, CPFX, and CP. One possible explanation for this is that these deletions have negative epistatic interactions with other mutations that contribute to the resistance acquisition to these drugs. To check this possibility, we added further genetic alterations to the Δ*arcA* and Δ*gutM* strains. We selected the genes *ompF* and *acrR* for deletion, as these are known as major contributors to the resistance acquisition to these drugs. Our previous laboratory evolution study showed that deletion strains conferring resistance to these drugs were commonly found to have mutations in these two genes^16^.

**Figure 4:**
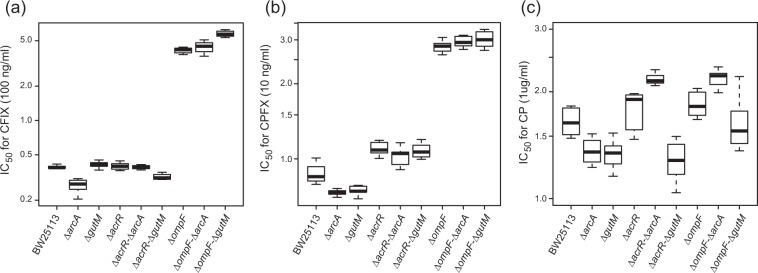
The IC_50_ values of single and double deletion strains. The boxplots represent IC_50_ values of 8 replicates for each strain. The IC_50_ values of 9 strains for (**a**) Cefixime (CFIX), (**b**) Ciprofloxacin (CPFX), and (**c**) Chloramphenicol (CP) are presented.

Figure 4 shows the IC_50_ values of the single and double mutant strains for the three drugs. Although the single mutant strains did not exhibit significant increases in CP resistance, the deletion of *ompF* significantly increased the resistance to CFIX and CPFX, and the deletion of *acrR* slightly increased the resistance to CPFX. Importantly, in the cases where the additional mutations led to increased resistance, the double deletion strains with Δ*arcA* and Δ*gutM* exhibited similar IC_50_ values to the single deletion strains of *ompF* and *acrR*. This suggests that there is little epistatic interaction between *arcA*/*gutM* deletions and the *ompF* and *acrR* mutations which are known to contribute to resistance to CFIX and CPFX. Of course, the possibility remains that other mutations beneficial to drug resistance have negative epistatic interactions with the deletion of *arcA*/*gutM*. However, these results indicate that at least some genes whose mutation is beneficial for drug resistance are present in Δ*arcA* and Δ*gutM* strains, even though they were not mutated during the laboratory evolution. In contrast, for the resistance acquisition to CP, the deletion of *gutM* did appear to exhibit negative epistatic interactions with the deletion of *ompF* and *acrR*, i.e., the double deletion strains Δ*acrR-*Δ*gutM* and Δ*ompF-*Δ*gutM* showed lower IC_50_ values than the single deletion strains Δ*acrR* and Δ*ompF* (Fig. 4c). This suggests that the observed suppression of resistance acquisition to CP in the deletion strain of *gutM* was caused by the epistatic interaction with *acrR* and *ompF* mutations. It should be noted that, the specific growth rates of Δ*acrR-*Δ*gutM* and Δ*ompF-*Δ*gutM* strains were 0.46 ± 0.01 and 0.37 ± 0.01 (1/h), respectively, under the condition without the addition of antibiotics. The latter was significantly smaller than that of the wild-type strain BW25113, which might suggest that the growth deficiency caused the observed epistatic interaction.

## Discussion

During antibiotic resistance evolution, various molecular mechanisms including gene regulation, membrane transport, and metabolic reactions need to be changed in a highly regulated manner to achieve higher fitness. Thus, to design drug treatments that suppress resistance evolution, we need to understand the complex interplay between the molecular mechanisms related to drug resistance. In this study, we screened for TFs whose deletion affects drug-resistance evolution of *E. coli* using high-throughput laboratory evolution combined with a single-gene deletion library. The results revealed that resistance evolution was significantly accelerated by the deletion of 21 genes and suppressed by the deletion of 42 genes, suggesting a complicated network between gene disruption and the mechanisms underlying resistance evolution.

We found that the *arcA* and *gutM* deletion strains exhibited significant suppression of resistance evolution to all three drugs tested, suggesting the involvement of these genes in the mechanisms of antibiotic resistance evolution. The *arcA* gene encodes the ArcA/B two-component system, which is a global regulator of gene expression under microaerobic and anaerobic growth conditions^25^. The ArcA/B system represses protein expression involved in aerobic respiration, such as those related to the tricarboxylic acid cycle, and activates gene expression involved in microaerobic or fermentative metabolism in response to redox conditions^26^. Thus, the deletion of *arcA* causes activation of aerobic respiration even in microaerobic or anaerobic conditions^27^. This metabolic change might contribute to the observed suppression of resistance evolution. The shift to aerobic metabolism caused by *arcA* deletion can enhance production of reactive oxygen species (ROS), and various studies have suggested that ROS production is involved in regulating the activation of antibiotic-mediated cell killing^28,29^. Thus, it is logical to postulate that evolution of antibiotic resistance favors anaerobic metabolism with relatively low ROS production. Our previous study^16^ demonstrated that the expression level of *arcA* was significantly increased in strains resistant to β-lactams, quinolones, and chloramphenicol obtained by laboratory evolution (Fig. S8), supporting the notion of a shift towards anaerobic metabolism. Furthermore, we quantified the intracellular ROS level by using 5-(and-6)-carboxy-2′,7′-dichlorodihydrofluorescein diacetate. The result is presented in Fig. S9, which also supported our hypothesis that the deletion of *arcA* results in higher ROS production. The activation of ROS production by *arcA* deletion could enhance the ROS-mediated effect of antibiotics, potentially suppressing resistance evolution.

The *gutM* gene encodes a DNA-binding transcription factor that regulates genes related to glucitol utilization^30^. So far, studies on the function of *gutM* have focused primarily on glucitol metabolism, therefore it is unclear how the deletion of this gene contributes to the suppression of resistance evolution. However, one study has suggested that *gutM* is involved in biofilm formation^31^, suggesting that it may have functions related to stress resistance. Further studies will be required to gain more mechanistic insight into how this gene affects antibiotic resistance evolution.

It should be noted that there was little epistatic interaction between the deletion of *arcA*/*gutM* and mutation of the genes *ompF* and *acrR*, which are known to be beneficial for resistance to CFIX and CPFX, suggesting that these deletion strains were unable to activate these beneficial mutations. The mechanism for this suppression of resistance acquisition by *arcA* and *gutM* deletions therefore remains unclear. One hypothesis is that the Δ*arcA* and Δ*gutM* strains transiently show sensitivity to these drugs when beneficial mutations are fixed, resulting in washing out these mutants. To verify this hypothesis, population dynamics under drug treatment could be performed at single-cell level, using microfluidic devices for example. Another possible explanation for the suppression of resistance evolution is a decreased mutation rate in these deletion strains. However, this seems unlikely for the Δ*arcA* strain, in which activation of the respiration pathway enhances the production of ROS, which is known to enhance mutation events via double-strand breaks.

In conclusion, the results of our systematic laboratory evolution study using single-gene deletion strains provided novel insights into targets for reducing the emergence of drug-resistant pathogens. For example, our results suggest that the combinatorial use of antibiotics and compounds that activate the respiration pathway, such as an ArcA phosphorylation inhibitor, might suppress resistance evolution to antibiotics. Although the use of the latter does not directly kill or inhibit the growth of pathogens, it could be beneficial by controlling resistance evolution to the antibiotic. Of course, the number of gene deletions to be analyzed was limited, and epistatic interactions among them remain largely unclear. However, our high-throughput system for laboratory evolution enabled us to quantitatively analyze the effect of gene perturbations on the resistance evolution to antibiotics, and such systematic analysis will be our future scope.

## Materials and Methods

### Strains and culture conditions

The *E. coli* BW25113 strain and the derivative Keio gene deletion library were obtained from the National BioResource Project (NIG, Japan). We selected strains with deletion of all TFs which regulate more than 2 genes according to RegulonDB^32^ (a list of the 173 selected genes is shown in Table S1). The laboratory evolution experiments were performed in modified M9 minimal medium^19^ with 5 g/L glucose as the carbon source. The strains were grown individually in 384-well plates (3680, Corning Inc., NY, USA) in 50 μl of M9 minimal medium with agitation at 300 rotations/min at 34 °C. All cultures were performed using an automated culture system consisting of a Biomek® NX span-8 laboratory automation workstation (Beckman Coulter, Tokyo, JP) in a sterile booth connected to a microplate reader (FilterMax F5; Molecular Devices, CA, USA), a shaker incubator (STX44; Liconic, Mauren, LI), and a microplate hotel (LPX220, Liconic, Mauren, LI). Before starting the laboratory evolution, the deletion strains were grown without the addition of antibiotics for at least more than 50 generations.

### Laboratory evolution under antibiotics

Eight independent cultures of each deletion strain were propagated in parallel in each concentration of a serial dilution of antibiotics (at slightly lower than the minimum inhibitory concentration). Laboratory evolution was performed at 12 different concentrations of antibiotics using 2^0.5^ (CFIX and CPFX) or 2^0.25^ (CP)-fold dilution steps. Antibiotic serial diluted plates were prepared using a Biomek® NX span-8 and Biomek® NX MC (Beckman Coulter, CA, USA). The growth of the cells was monitored daily by measuring the OD_620_ of each well using a microplate reader (FilterMax F5, Molecular Devices, CA, USA). We defined wells with OD_620_ greater than 0.1 as viable wells. Cells calculated to yield an initial OD_620_ of 3 × 10^−4^ (corresponding to approximately 1 × 10^3^ cells per well), were transferred from the viable well with the highest drug concentration to new plates with fresh medium and various concentrations of antibiotics.

### Data analysis

To obtain the IC_50_ values, the OD_620_ values for the dose-response series were fitted to the following sigmoidal model:

$$f(x)=\frac{a-c}{1+\exp \{b\times (\log \,x-{{\rm{logIC}}}_{50})\}}+c$$

where *x* and *f*(*x*) represent the concentration of antibiotics and the observed OD_620_ values, respectively, and *a*, *b*, and c are fitting parameters. The fitting was performed by a greedy genetic algorithm with a custom-made C program. All statistical analyses shown in Fig. 3 were performed in R programming language.

### Construction of double-deletion mutants

Using Keio single-gene deletion strains as the parental strain, double-deletion mutants were constructed by multiplexed automated genome engineering (MAGE) using a pORTMAGE-4 vector^33^. The plasmid pORTMAGE-4 was a gift from Csaba Pál (Addgene plasmid #72679). Sequences of MAGE oligos were created using the MODEST tool^34^. An NheI site containing a TAG stop codon and one base frame-shift mutation was inserted into the ORF region of *ompF* or *acrR* to construct double-deletion mutants following the procedure reported previously^33^. Briefly, the MAGE oligo was electroporated into the parental strain and inserted into the target locus by λ-Red recombinase on the plasmid pORTMAGE-4. The genome modifications were verified by colony PCR and Sanger sequencing of the PCR products. The sequences of oligos and primers used in this study are shown in Table S3.

### Reactive oxygen species assay

To assess the cellular reactive oxygen species (ROS), 5-(and-6)-carboxy-2′,7′-dichlorodihydrofluorescein diacetate (carboxy-H_2_DCFDA) (C400, Invitrogen Co., CA, USA) was used as a fluorescent indicator of the intra-cellular ROS level. Each overnight culture was added to 200 μl of fresh M9 medium in the 96-well glass-bottom plate (265300, Thermo Fisher Scientific Inc., MA, USA) and cultivated at 34 °C. The cells in the exponential growth phase were harvested by centrifugation and washed by pre-warmed PBS. As per the manufacturer’s instruction, H_2_O_2_ (100 μM final concentration) was added to one set of control cells and incubated at 34 °C for 30 min. Before centrifugation as the positive control for ROS production. After centrifugation, carboxy-H_2_DCFDA was added to one set of samples (10 μM final concentration) and incubated at 34 °C for 30 min. The other set of samples that were incubated without adding carboxy-H_2_DCFDA were used for the measurement of autofluorescence of the cell. The cells were centrifuged and suspended by PBS to remove the extracellular carboxy-H_2_DCFDA. The cell density (OD_600_) and fluorescence (Abs_535_) were measured by using the microplate reader (1420 ARVO, PerkinElmer Inc., MA, USA). Specific fluorescence was calculated as follows.

$${\rm{S}}{\rm{p}}{\rm{e}}{\rm{c}}{\rm{i}}{\rm{f}}{\rm{i}}{\rm{c}}\,{\rm{F}}{\rm{l}}{\rm{u}}{\rm{o}}{\rm{r}}{\rm{e}}{\rm{s}}{\rm{c}}{\rm{e}}{\rm{n}}{\rm{c}}{\rm{e}}=\frac{{{\rm{A}}{\rm{b}}{\rm{s}}}_{535}({\rm{c}}{\rm{a}}{\rm{r}}{\rm{b}}{\rm{o}}{\rm{x}}{\rm{y}} \mbox{-} {{\rm{H}}}_{2}{\rm{D}}{\rm{C}}{\rm{F}}{\rm{D}}{\rm{A}}[+])}{{{\rm{O}}{\rm{D}}}_{600}({\rm{c}}{\rm{a}}{\rm{r}}{\rm{b}}{\rm{o}}{\rm{x}}{\rm{y}} \mbox{-} {{\rm{H}}}_{2}{\rm{D}}{\rm{C}}{\rm{F}}{\rm{D}}{\rm{A}}[+])}-\frac{{{\rm{A}}{\rm{b}}{\rm{s}}}_{535}({\rm{c}}{\rm{a}}{\rm{r}}{\rm{b}}{\rm{o}}{\rm{x}}{\rm{y}} \mbox{-} {{\rm{H}}}_{2}{\rm{D}}{\rm{C}}{\rm{F}}{\rm{D}}{\rm{A}}[-])}{{{\rm{O}}{\rm{D}}}_{600}({\rm{c}}{\rm{a}}{\rm{r}}{\rm{b}}{\rm{o}}{\rm{x}}{\rm{y}} \mbox{-} {{\rm{H}}}_{2}{\rm{D}}{\rm{C}}{\rm{F}}{\rm{D}}{\rm{A}}[-])}$$

## Supplementary information

Supplementary Figures.

Table S1: List of genes.

Table S2: Resistance level of deletion strains.

Table S3: Primer information.
