# Supplementary material for: Suppression of antibiotic resistance evolution by single-gene deletion
Source: Sci Rep. 2020 Mar 6;10:4178. doi: 10.1038/s41598-020-60663-6 (PMC7060189; doi:10.1038/s41598-020-60663-6)
Supplement: Supplementary file 1 — Supplementary Figures. [file 41598_2020_60663_MOESM1_ESM.pdf]

Supplementary Information

Suppression of antibiotic resistance evolution by single-gene deletion

Takaaki Horinouchi[1], Tomoya Maeda[1], Hazuki Kotani[1], Chikara Furusawa[1,2*]

[1]Center for Biosystems Dynamics Research, RIKEN, 6-2-3 Furuedai, Suita, Osaka 565-0874, Japan
[2]Universal Biology Institute, The University of Tokyo, 7-3-1 Hongo, Tokyo 113-0033, Japan

*Correspondence:
Chikara Furusawa
Center for Biosystems Dynamics Research, RIKEN, 6-2-3 Furuedai, Suita, Osaka 565-0874, Japan
Tel: +81-6-6155-0489
E-mail: chikara.furusawa@riken.jp

Legends of Supplementary Figures

Figure S1. The time courses of 173 deletion strains of transcription factors under Cefixime (CFIX).

Figure S2. The time courses of 173 deletion strains of transcription factors under Ciprofloxacin (CPFX).

Figure S3. The time courses of 173 deletion strains of transcription factors under Chloramphenicol (CP).

Figure S4. The time courses of IC_{50} for *ΔrpoS* in the laboratory evolution with the three indicated drugs (Cefixime; CFIX, Ciprofloxacin; CPFX, Chloramphenicol; CP).

Figure S5. The relationship between growth rate and resistance evolution. The horizontal and vertical axes represent the maximal growth rate and the final cell concentration (OD_{620}) of 173 deletion strains of transcription factors, respectively (obtained from a previous study[35]). The green triangles and blue squares show the deletion strains exhibiting significantly higher and lower IC_{50} values than the wild-type strain BW25113 (without deletion) in this study (FDR<0.1), respectively. The results for (a) Cefixime (CFIX), (b) Ciprofloxacin (CPFX), and (c) Chloramphenicol (CP) are presented.

Figure S6. The relationship between mutation rate and resistance evolution. The data of the mutation rates of 12 TF deletion strains were obtained by the previous study[24], which was obtained by the frequency of Lac^+ mutant.

Figure S7. Venn diagram representing genes whose deletion resulted in significantly higher IC_{50} values for the three indicated drugs (Cefixime; CFIX, Ciprofloxacin; CPFX, Chloramphenicol; CP) on the last day of laboratory evolution in comparison with the wild-type strain BW25113 (without gene deletion). The genes with FDR (calculated by Benjamini-Hochberg method) < 0.1 are presented.

Figure S8. Expression levels of *arcA* in antibiotic-resistant strains. The expression levels were taken from a previous study[16], in which the resistant strains were obtained by 90 days of laboratory evolution by a similar procedure to the present study. The data are categorized into the drug groups; β-lactam (Cefixime and Cefoperazone), quinolone (Ciprofloxacin and Enoxacin), and Chloramphenicol. The log_{10}-transformed expression ratios to the ancestor strains are presented.

Figure S9. Intracellular ROS levels of *ΔarcA*. quantified by using carboxy-H_2DCFDA. For the control, the results of wild-type strain BW25113 with and without the addition of 100 μM H_2O2 to the medium are also presented. The error bars represent the standard deviation of triplicate measurements.

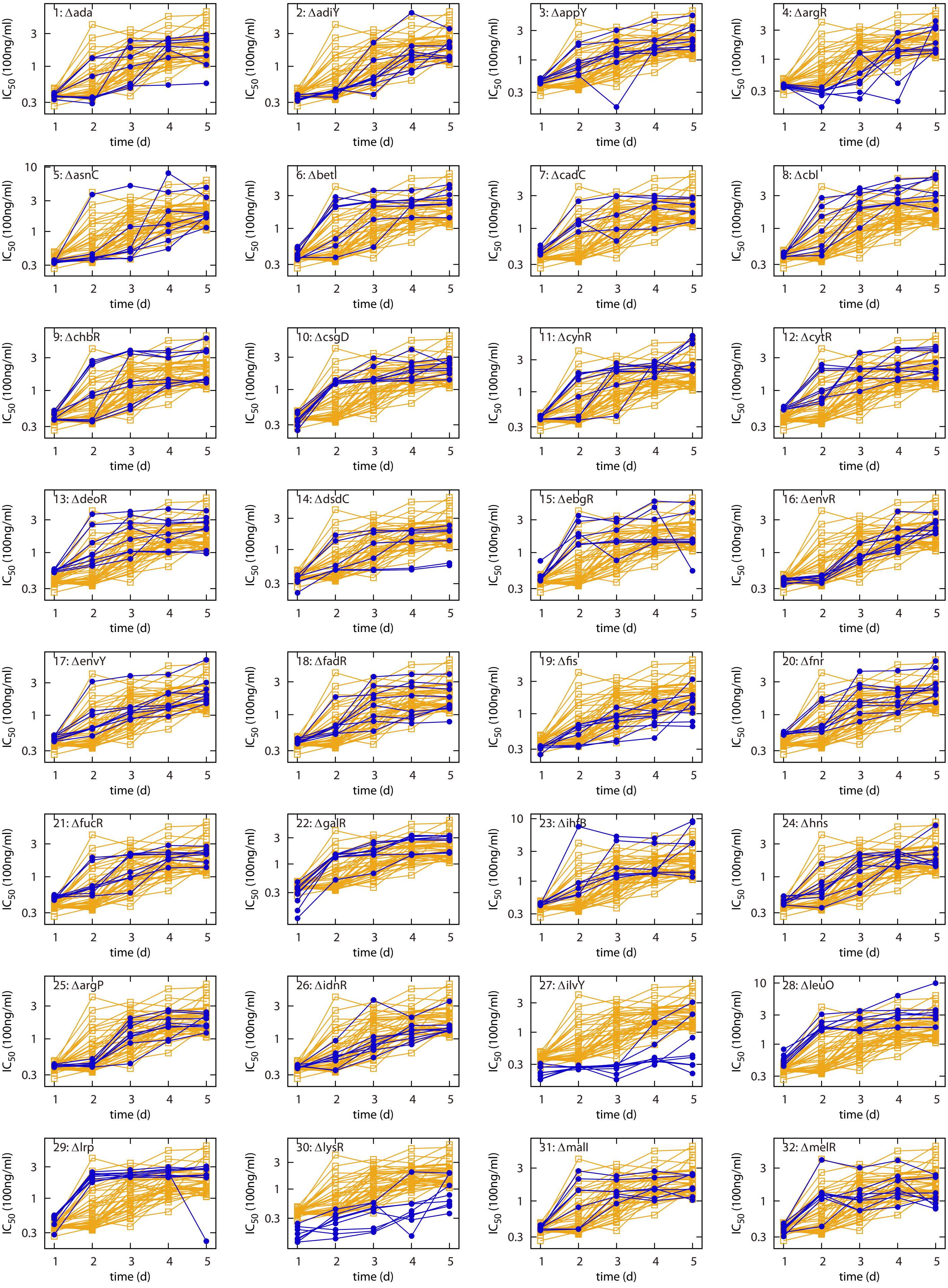

Horinouchi et al, Figure S1

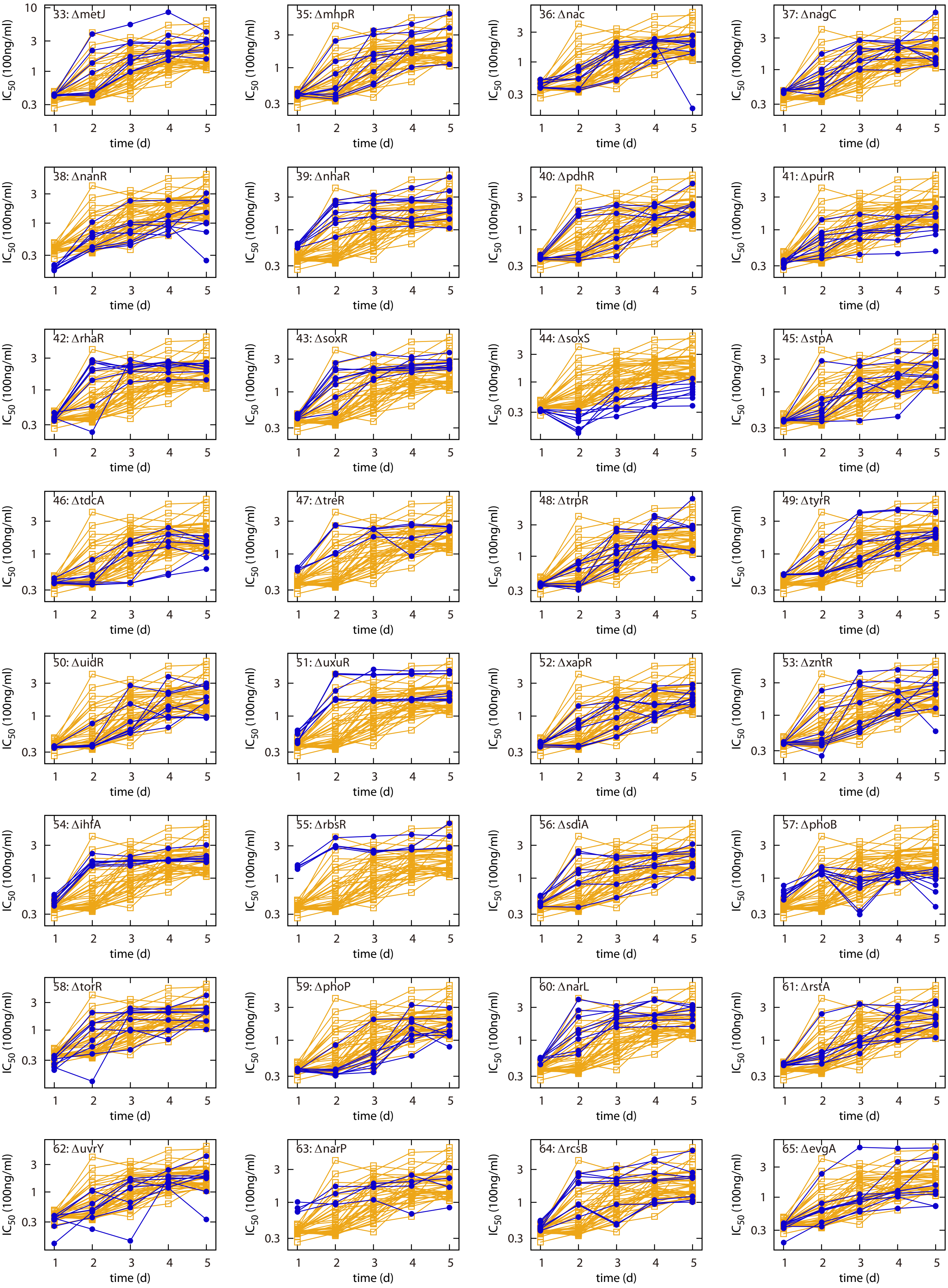

Horinouchi et al, Figure S1 (continued)

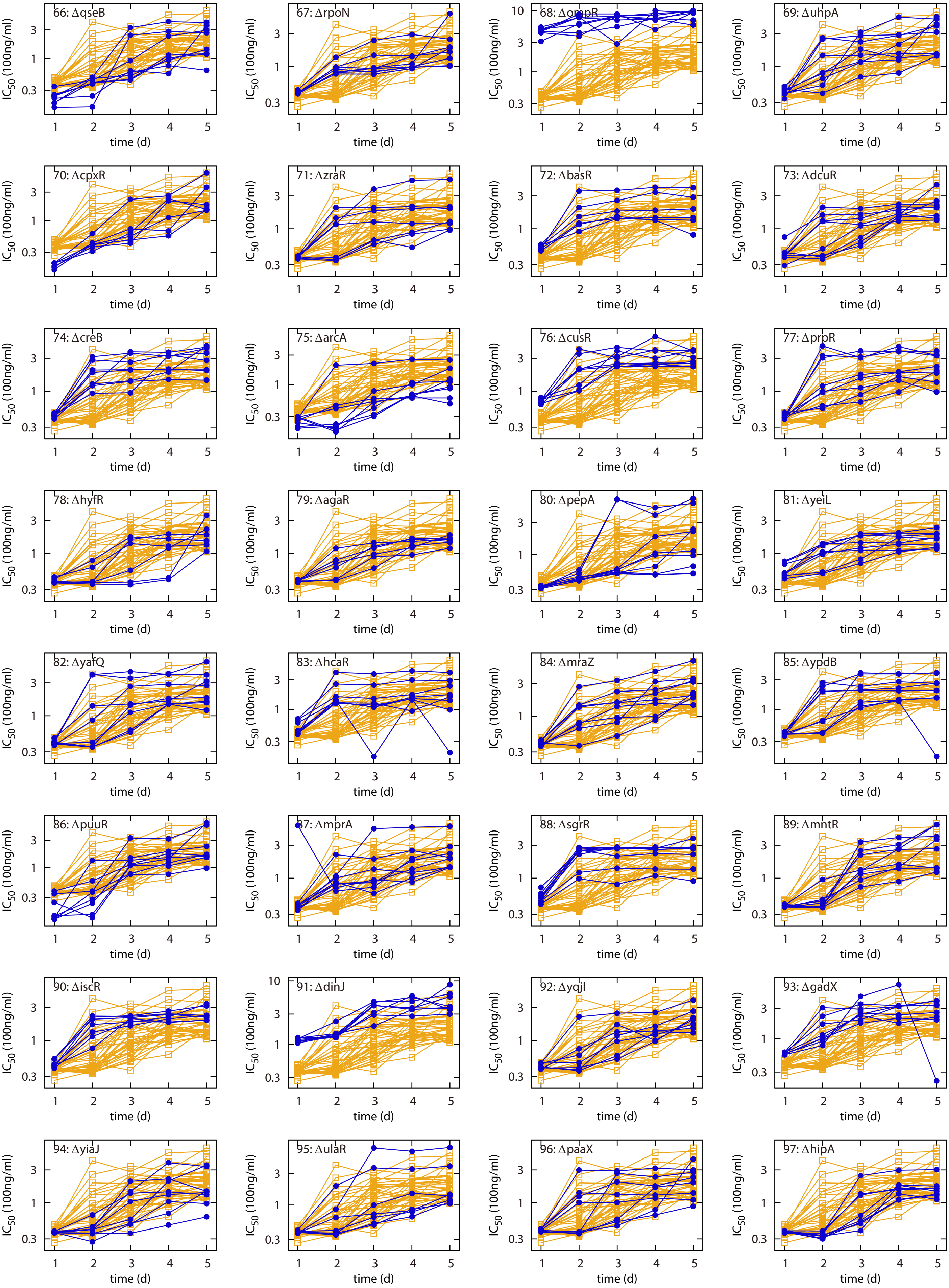

Horinouchi et al, Figure S1 (continued)

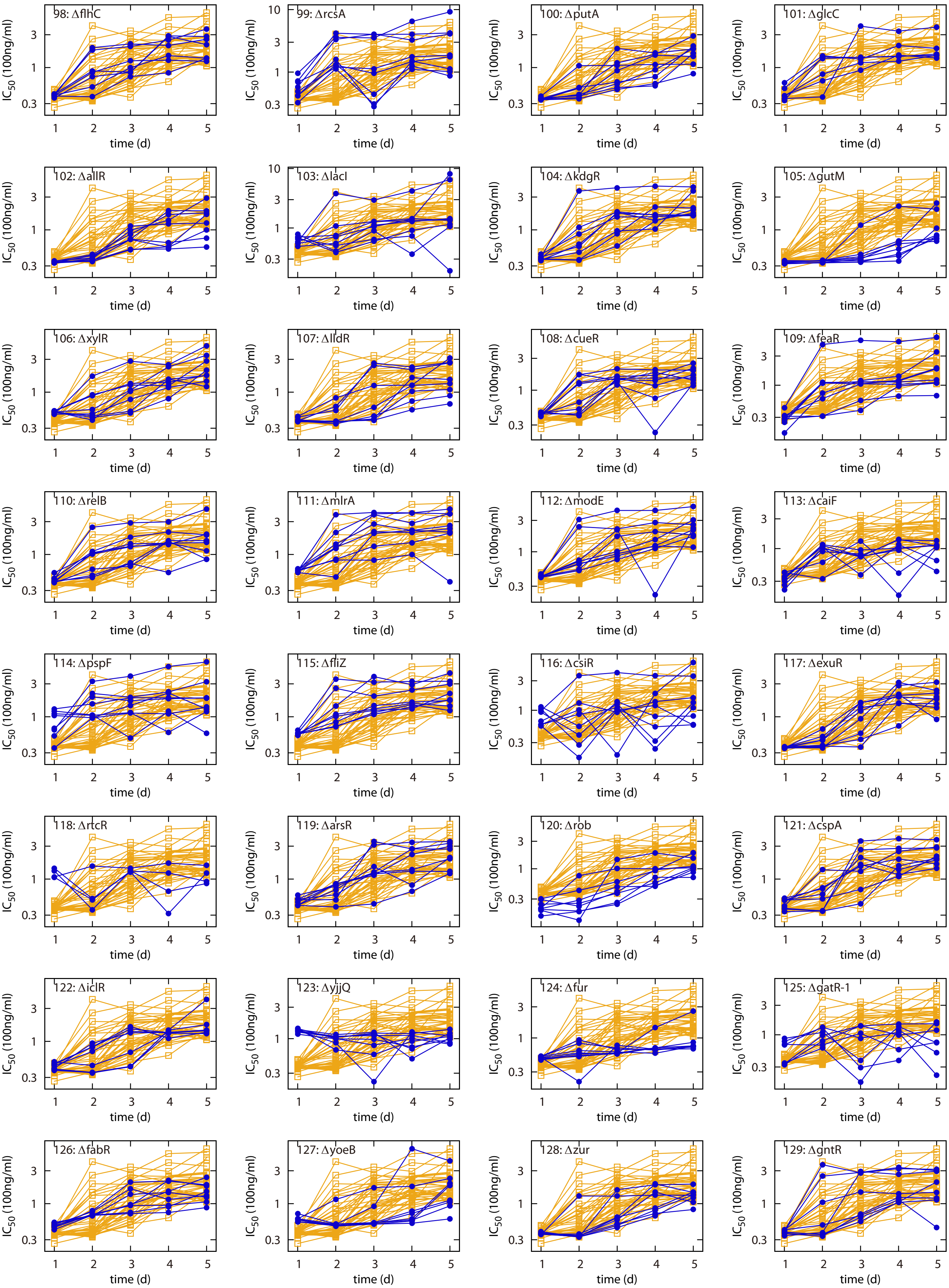

Horinouchi et al, Figure S1 (continued)

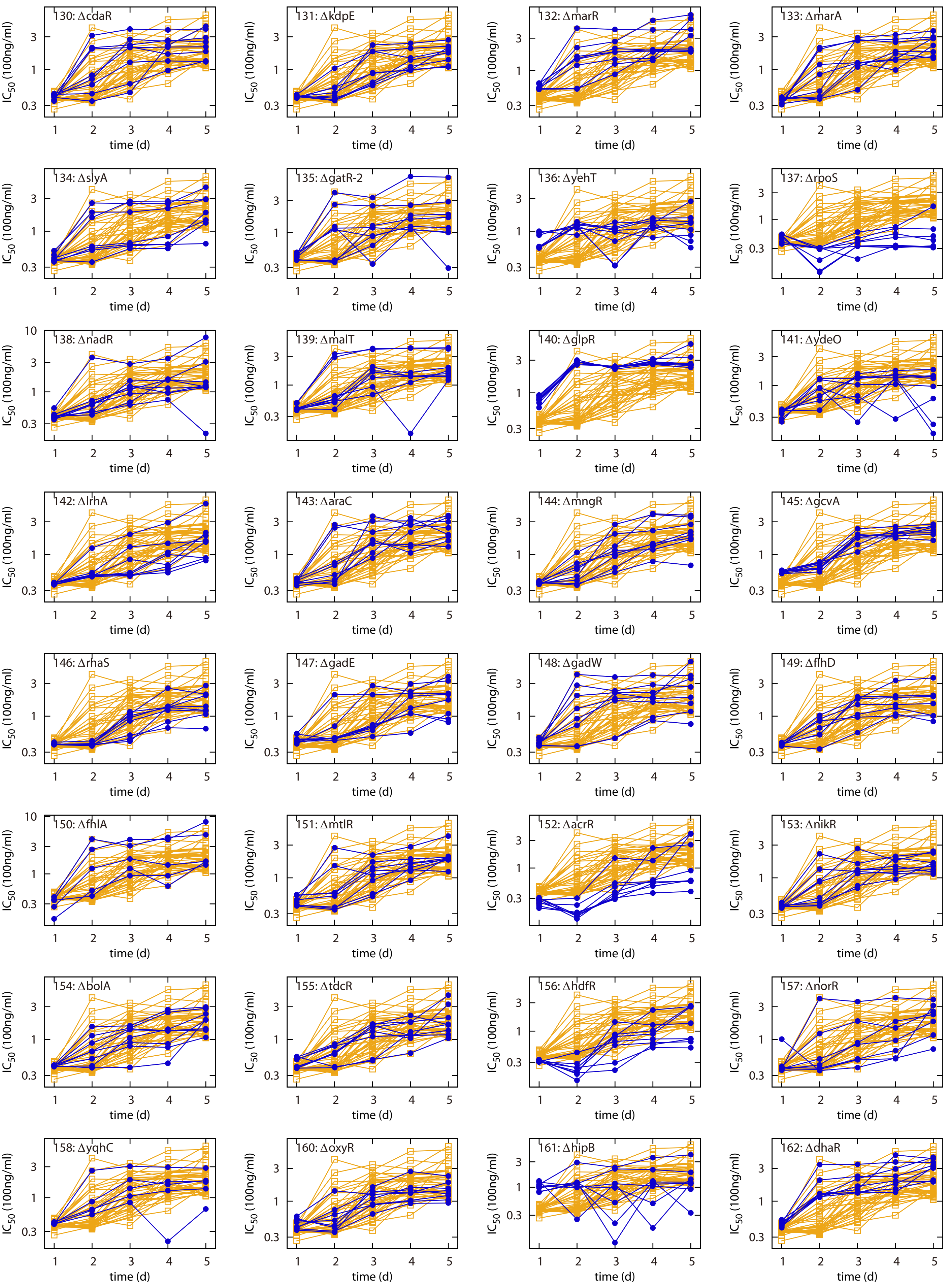

Horinouchi et al, Figure S1 (continued)

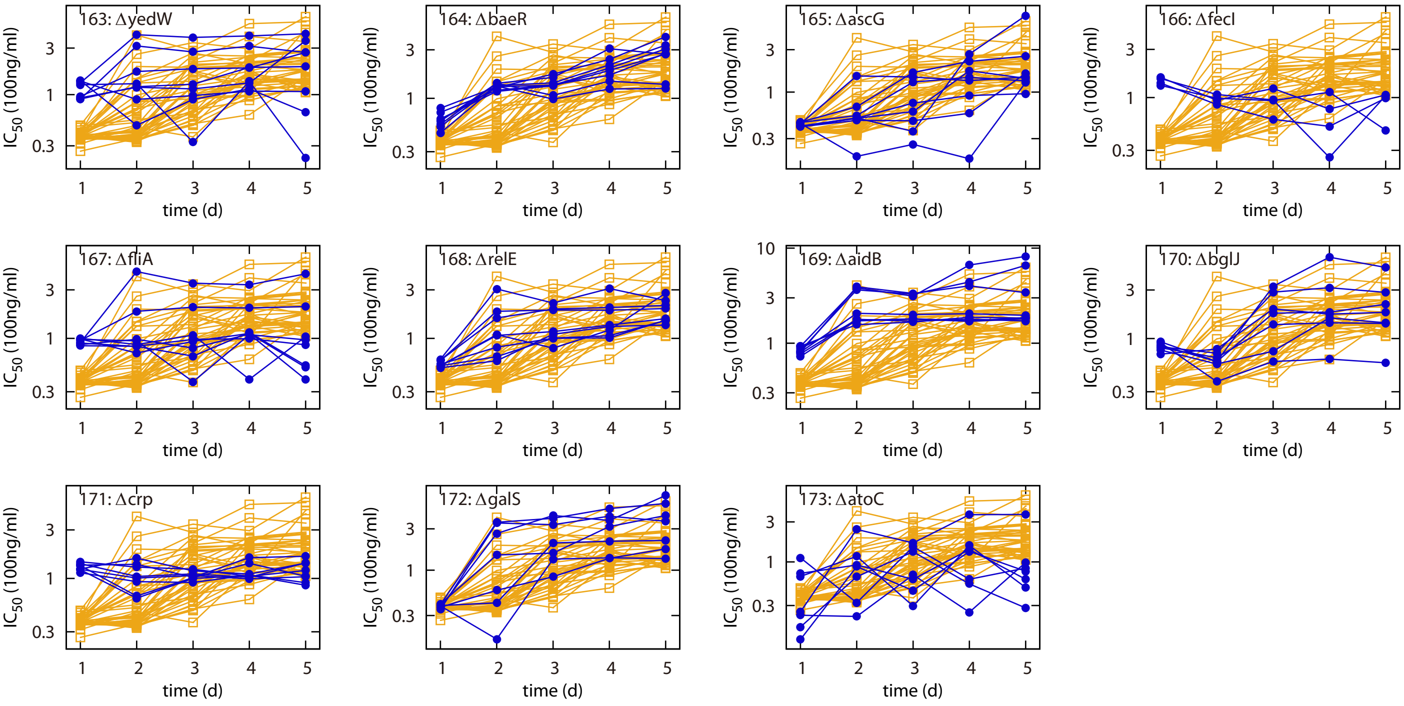

Horinouchi et al, Figure S1 (continued)

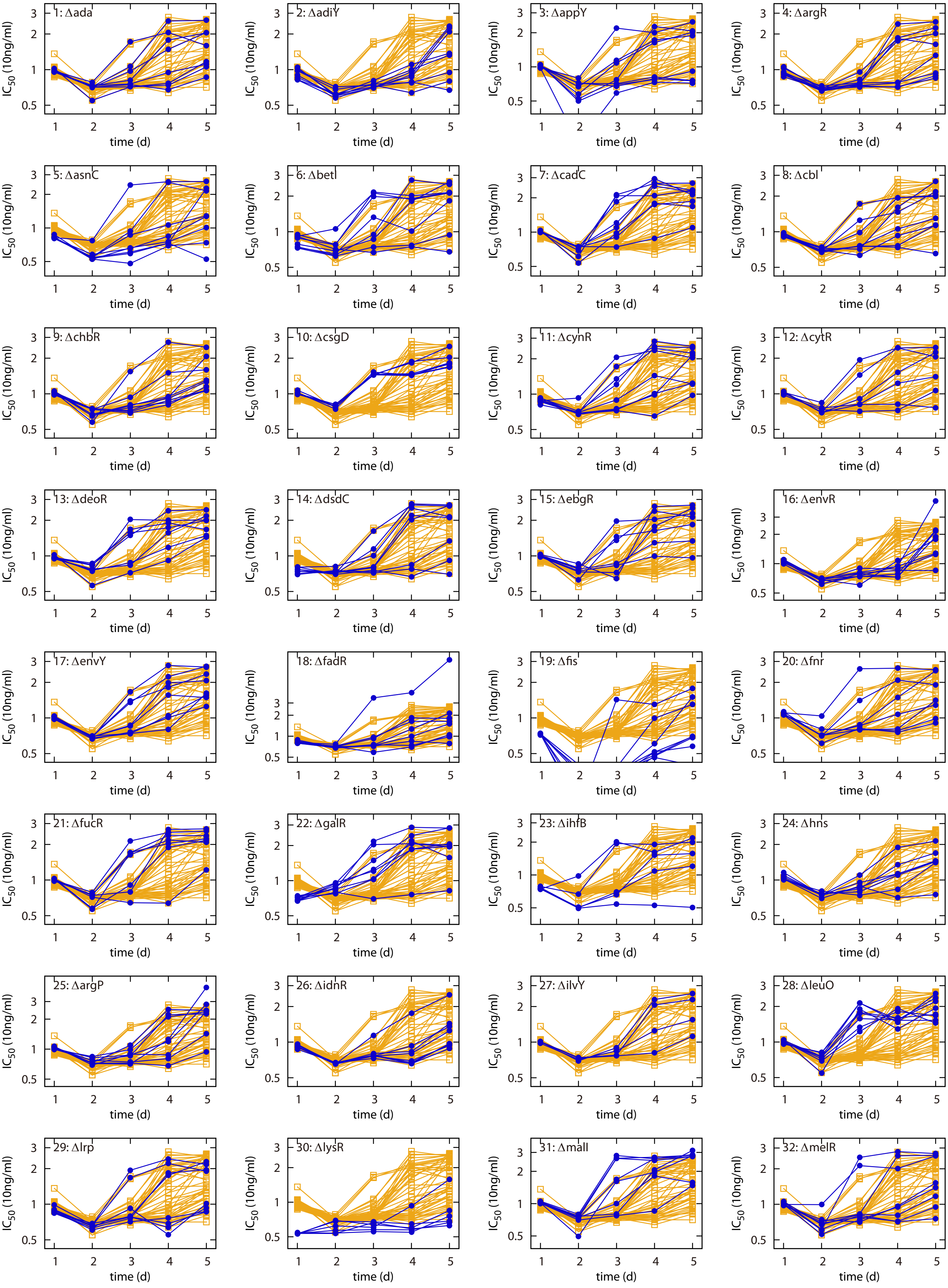

Horinouchi et al, Figure S2

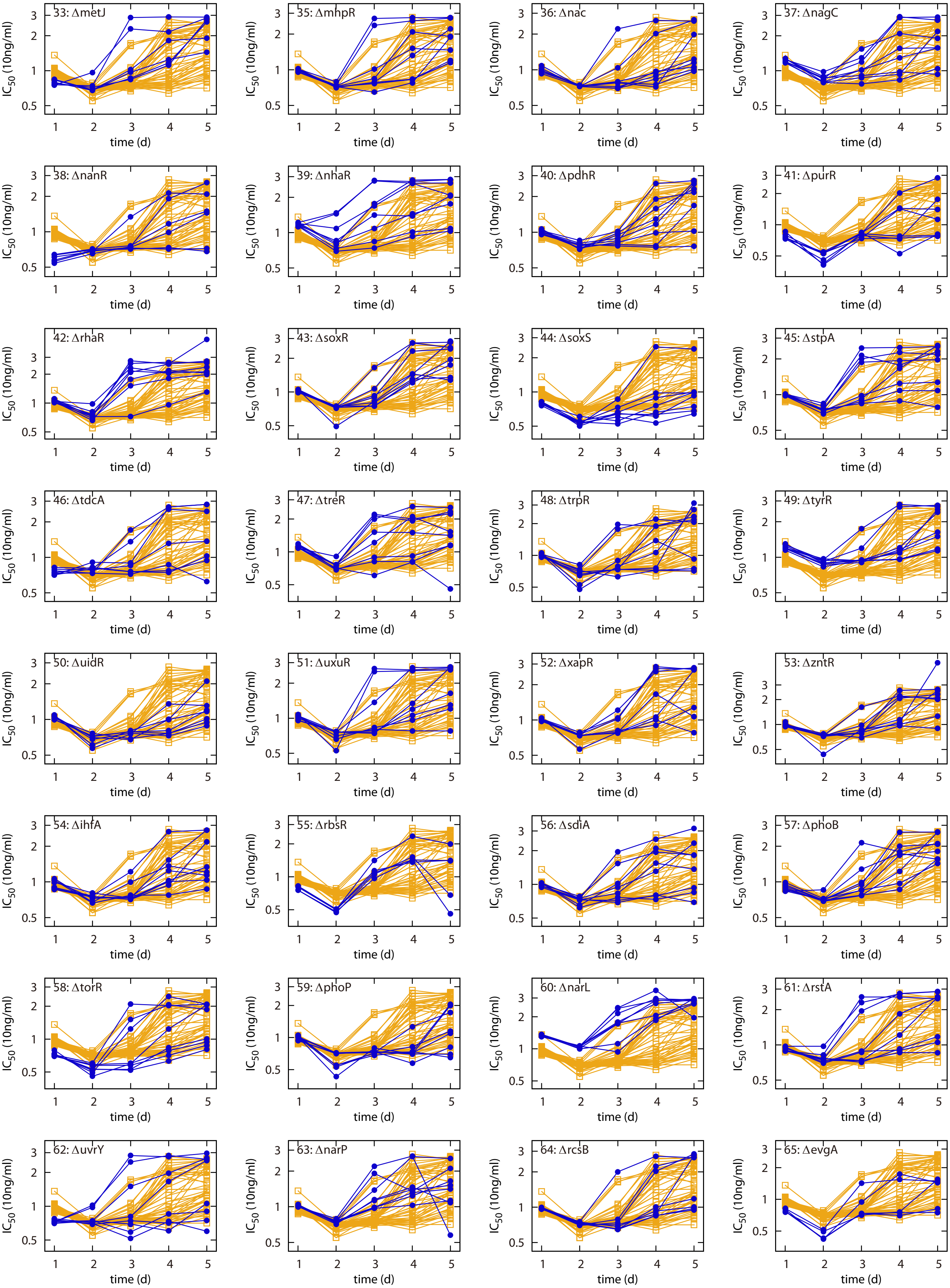

Horinouchi et al, Figure S2 (continued)

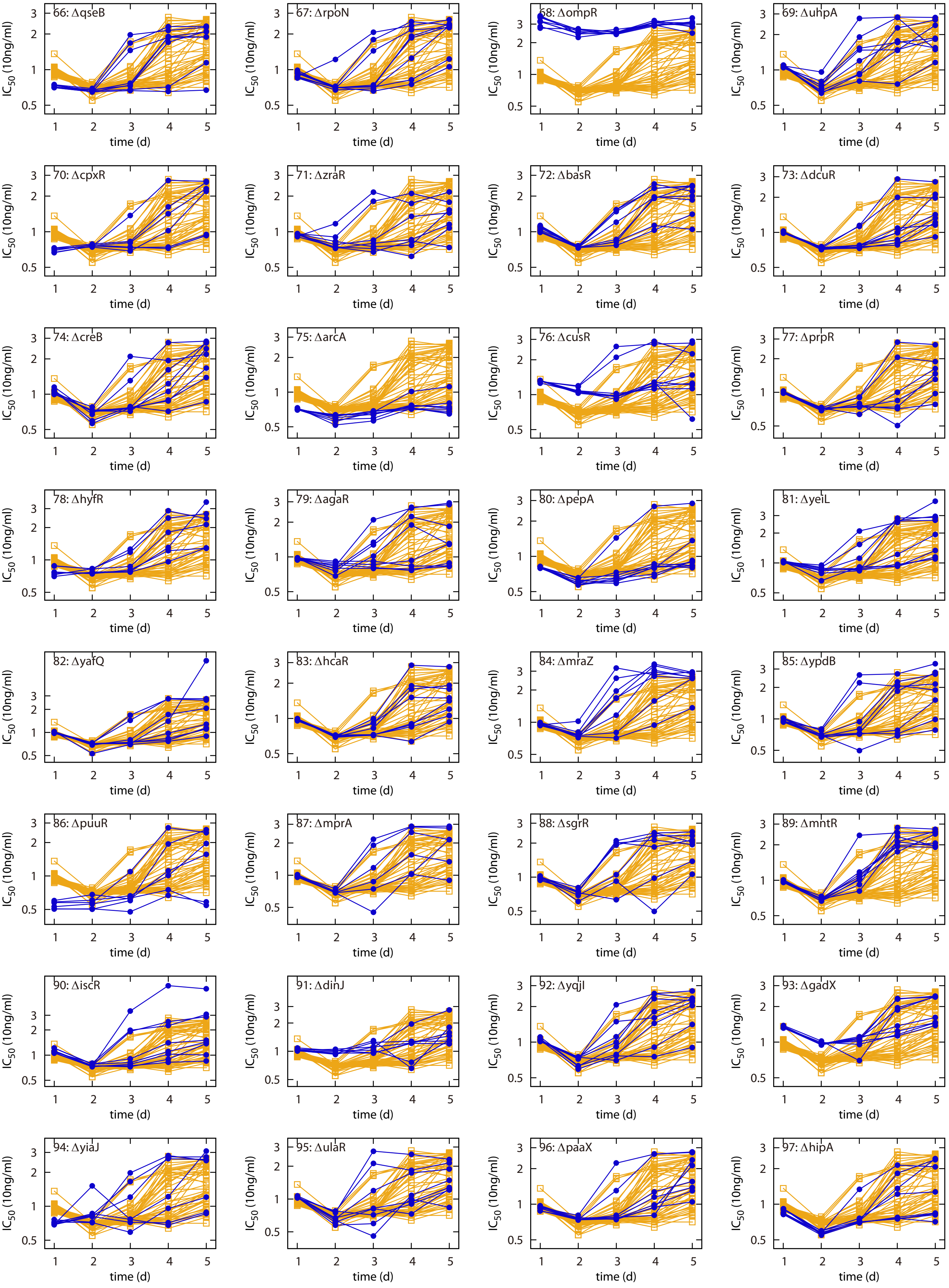

Horinouchi et al, Figure S2 (continued)

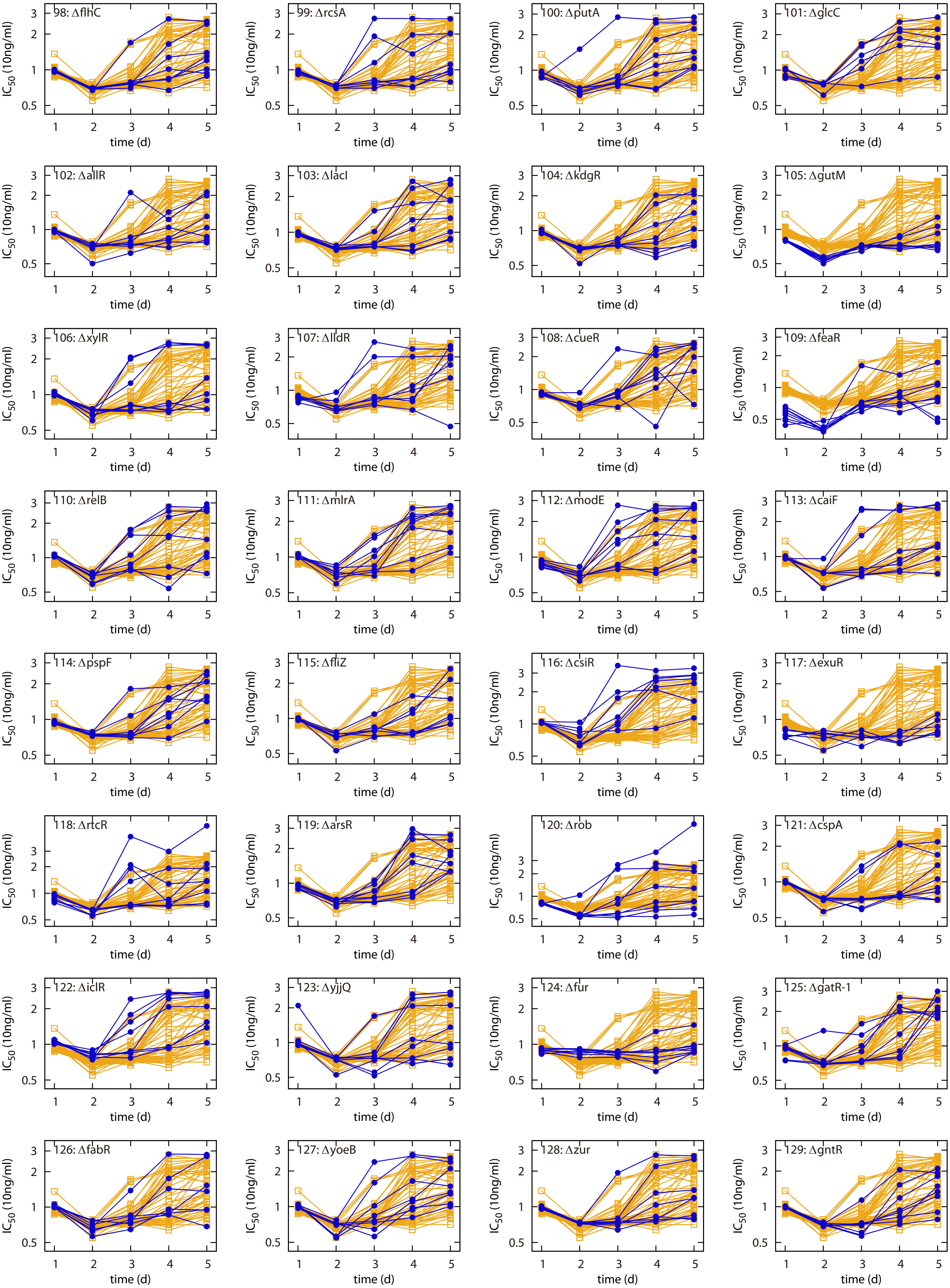

Horinouchi et al, Figure S2 (continued)

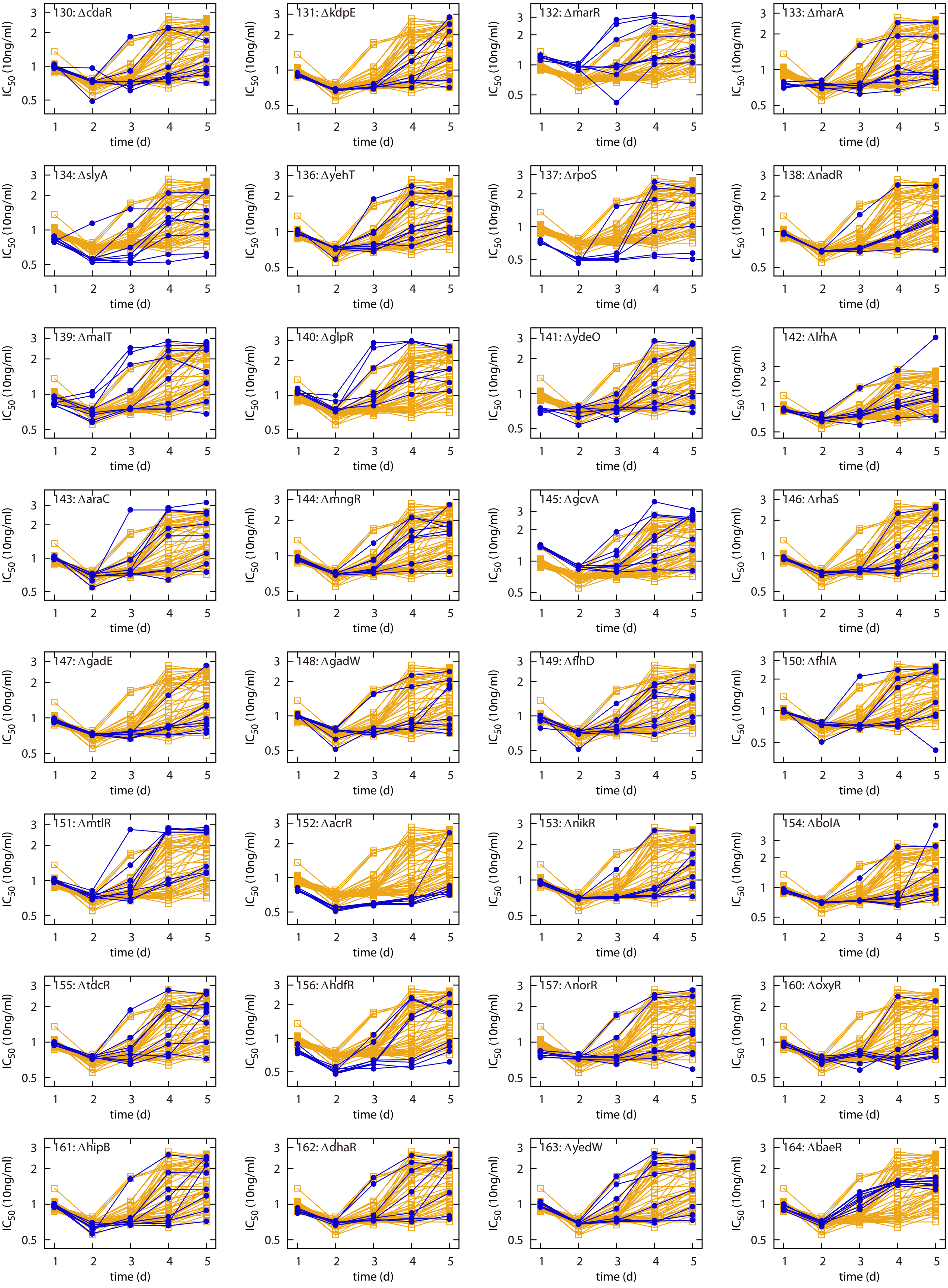

Horinouchi et al, Figure S2 (continued)

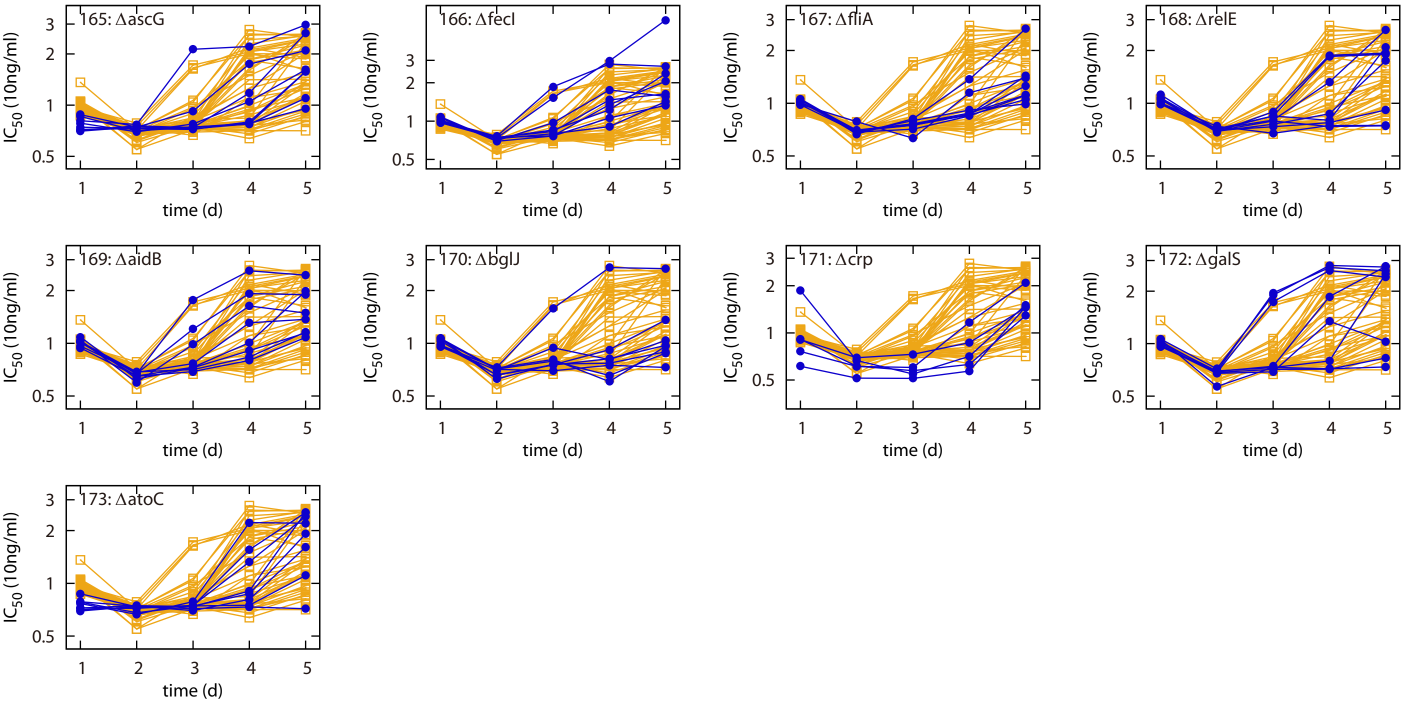

Horinouchi et al, Figure S2 (continued)

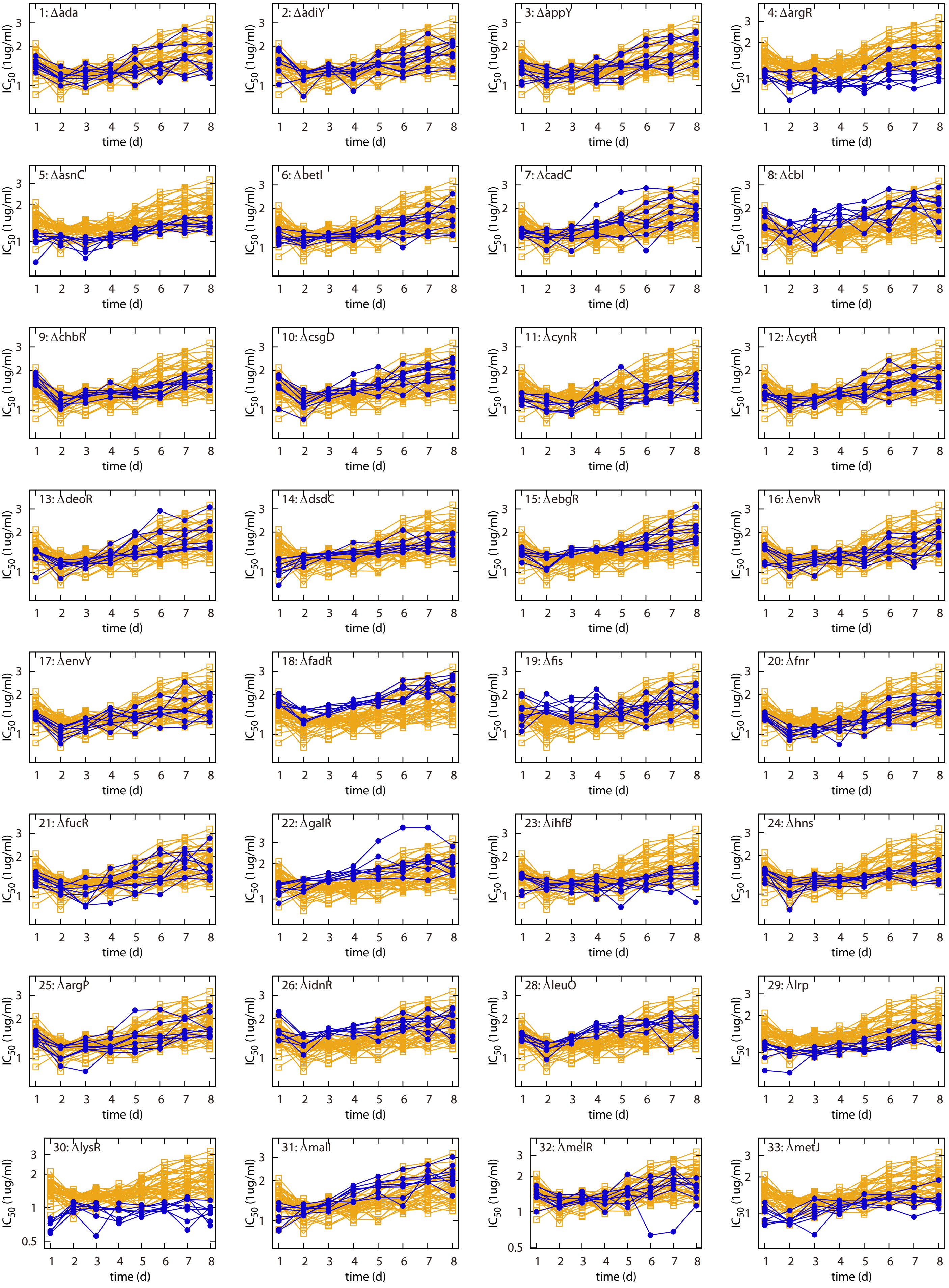

Horinouchi et al, Figure S3

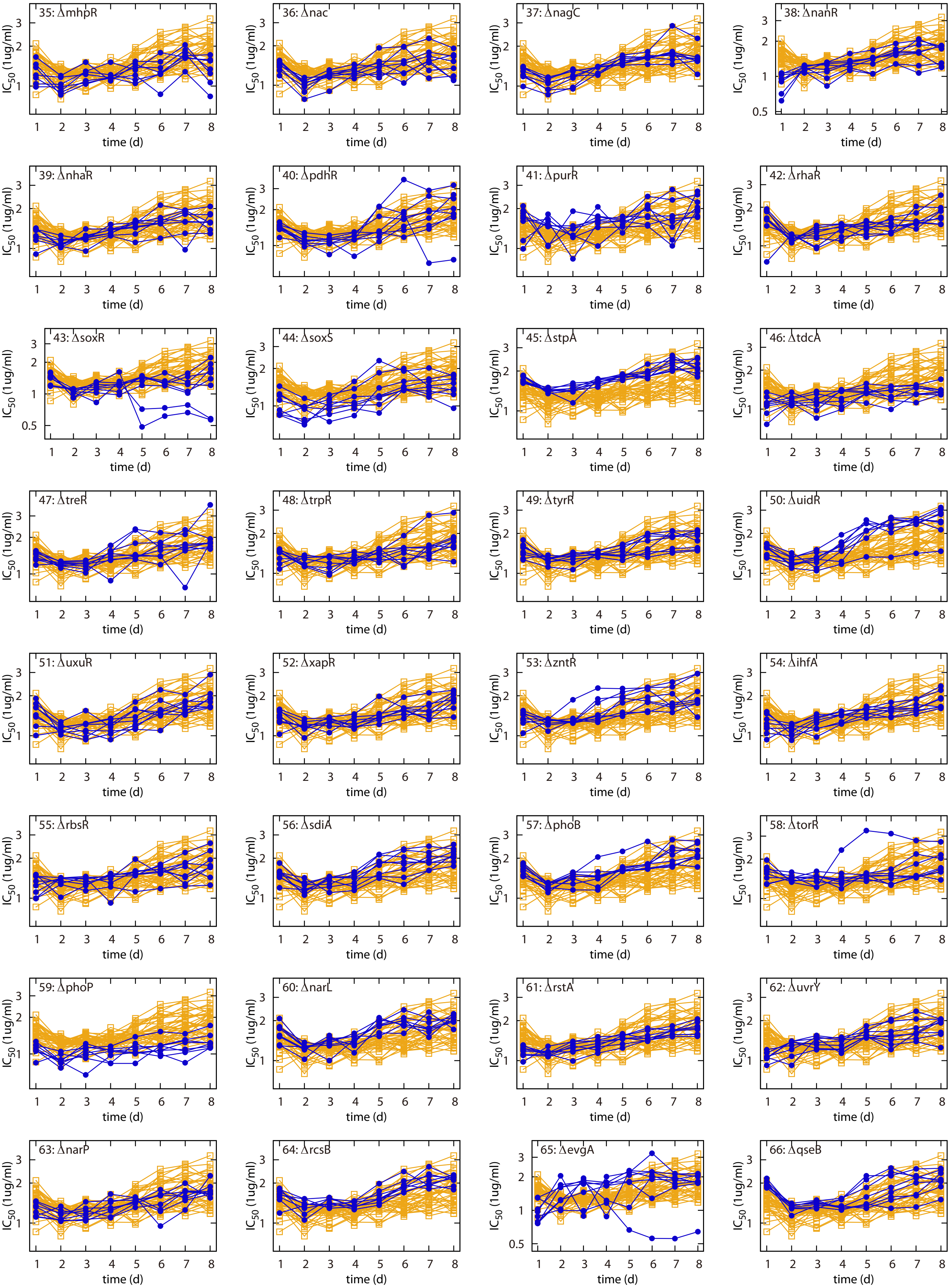

Horinouchi et al, Figure S3 (continued)

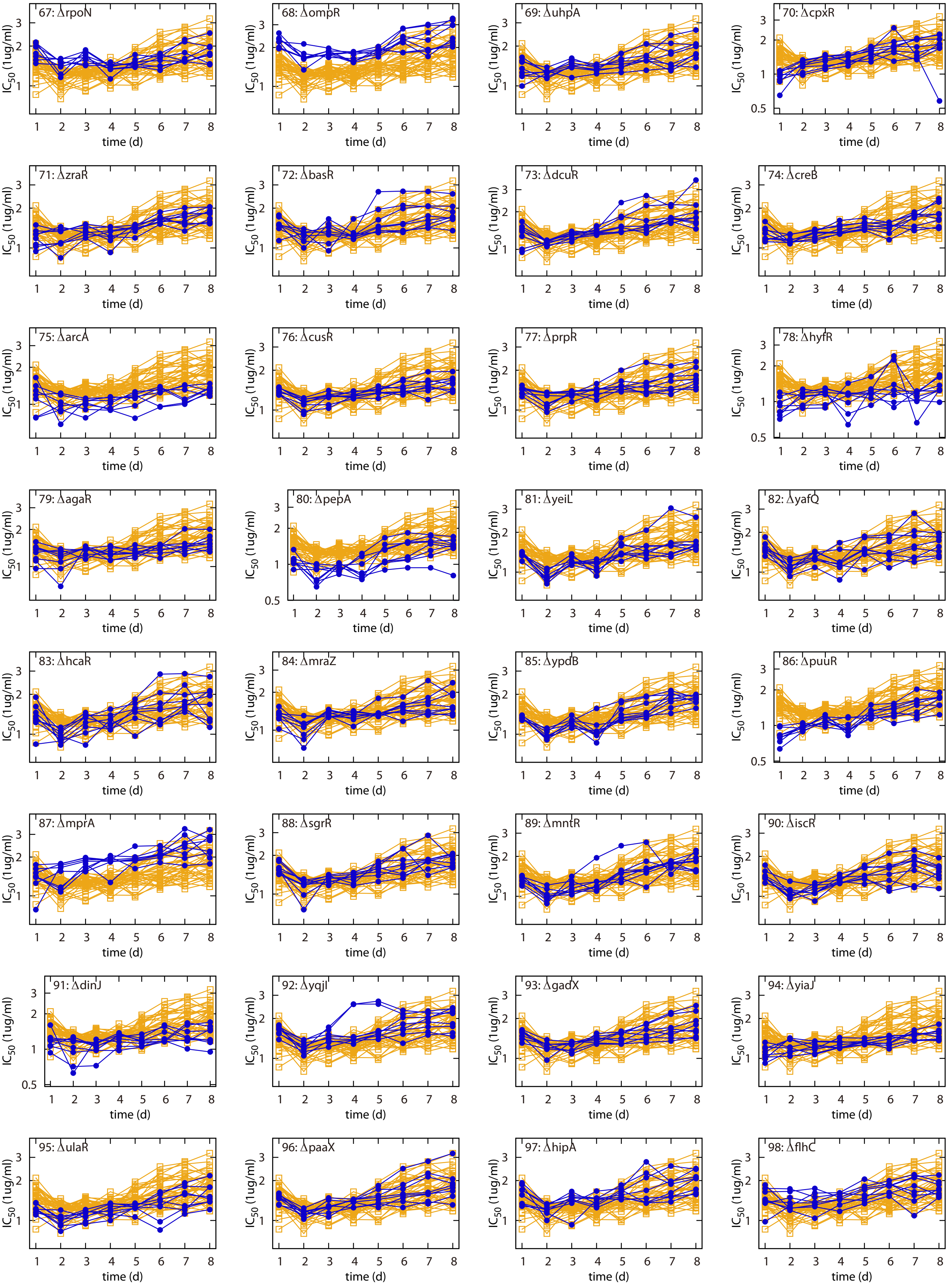

Horinouchi et al, Figure S3 (continued)

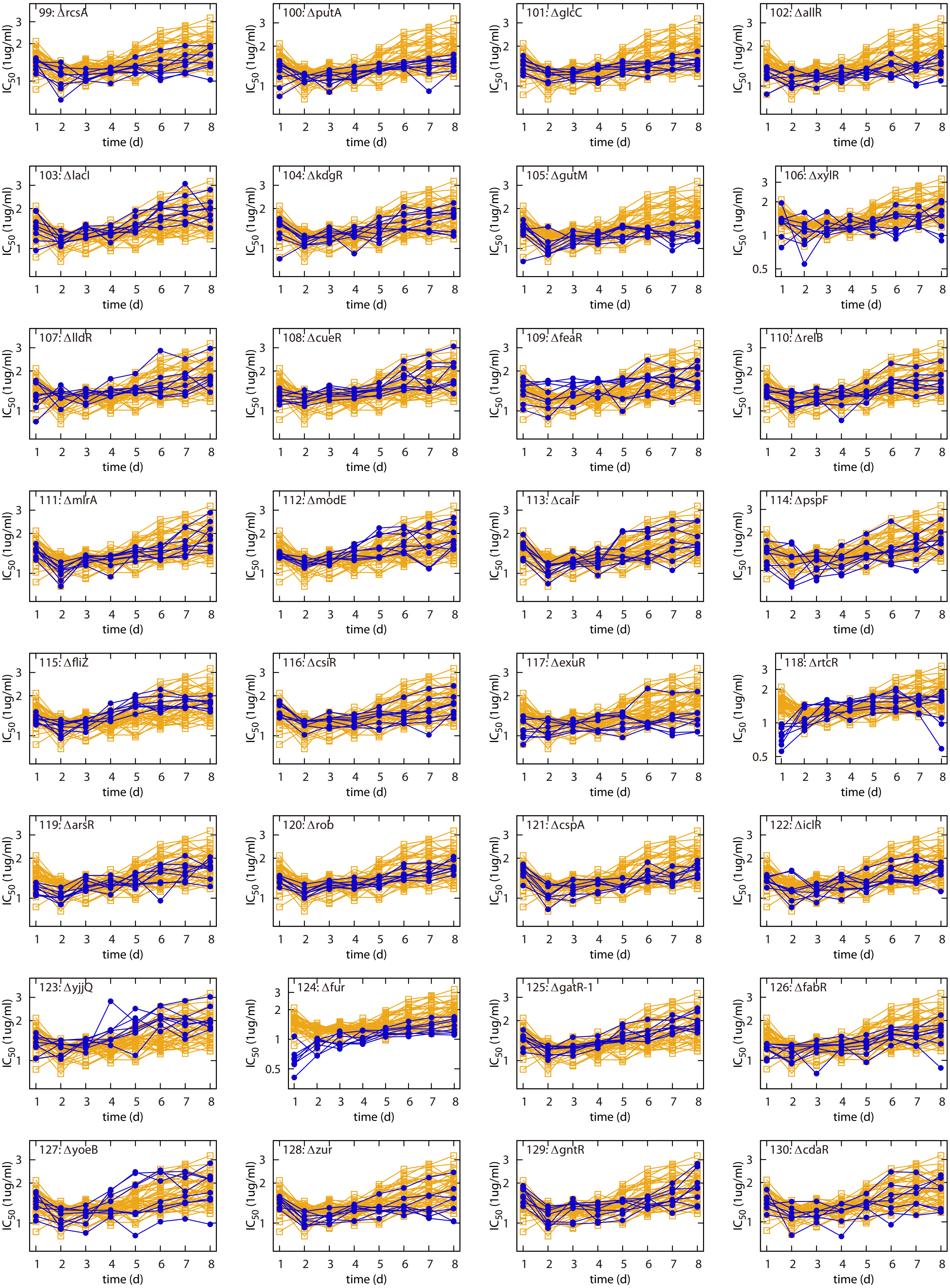

Horinouchi et al, Figure S3 (continued)

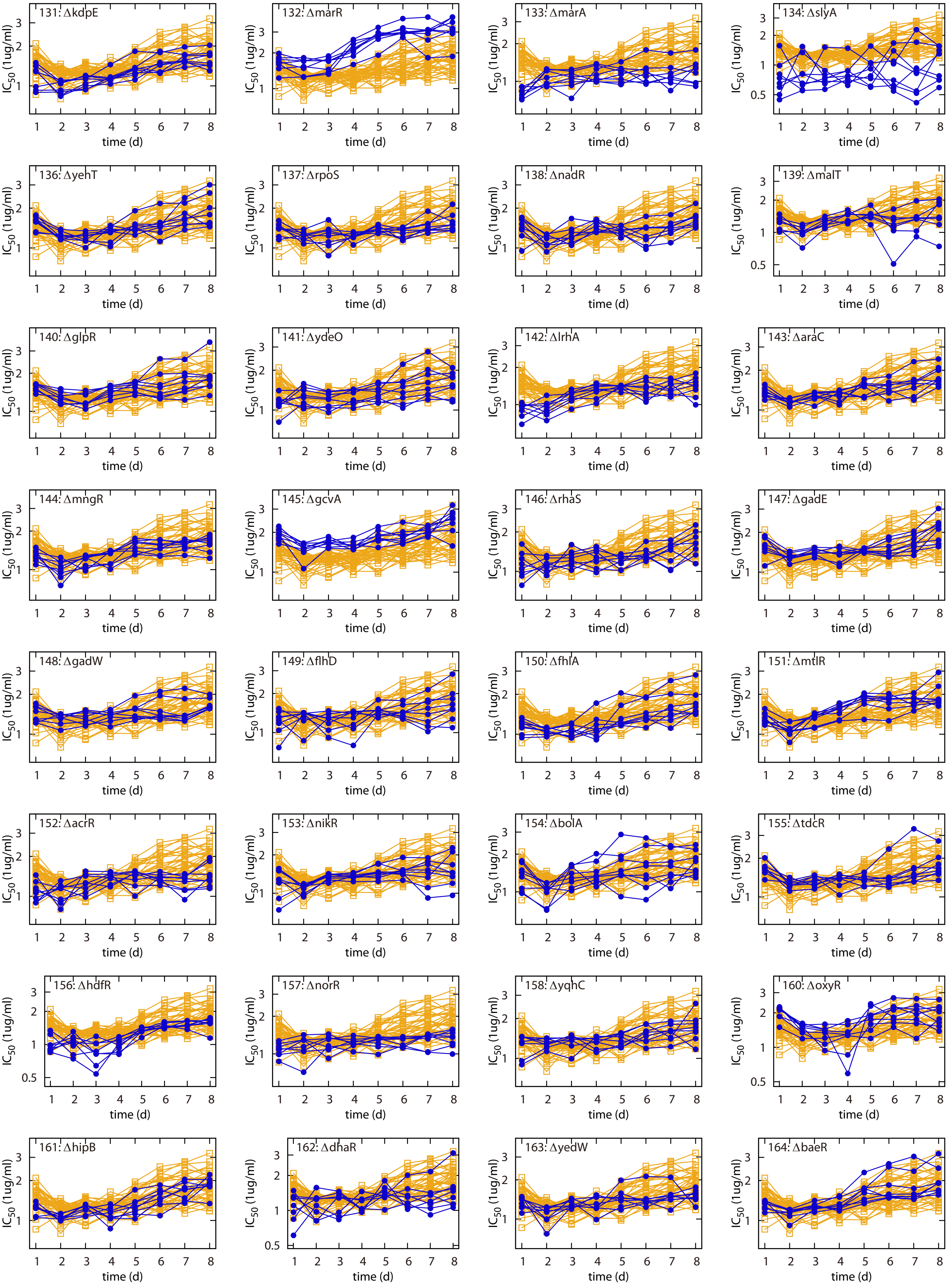

Horinouchi et al, Figure S3 (continued)

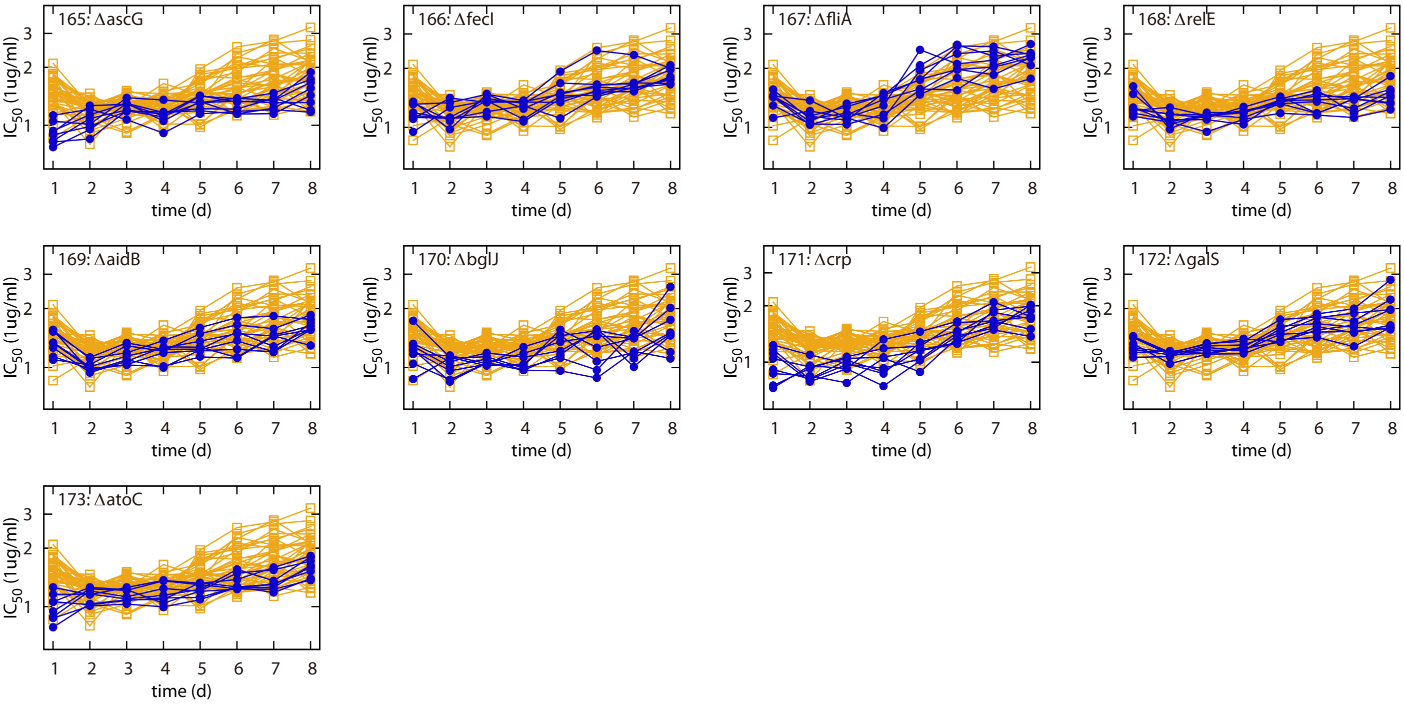

Horinouchi et al, Figure S3 (continued)

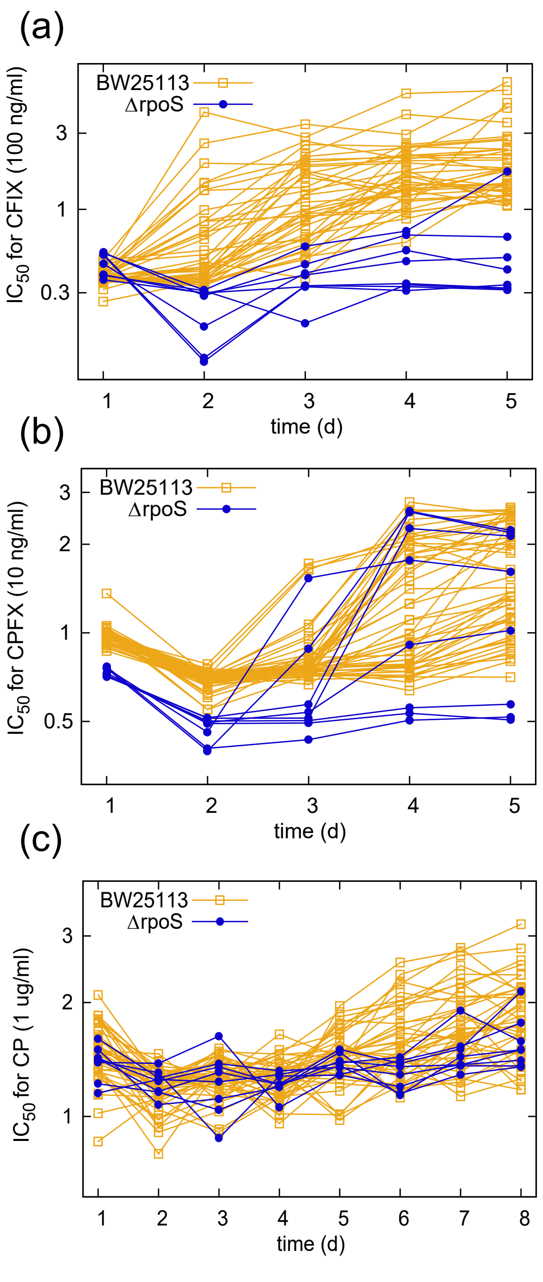

Horinouchi et al, Figure S4

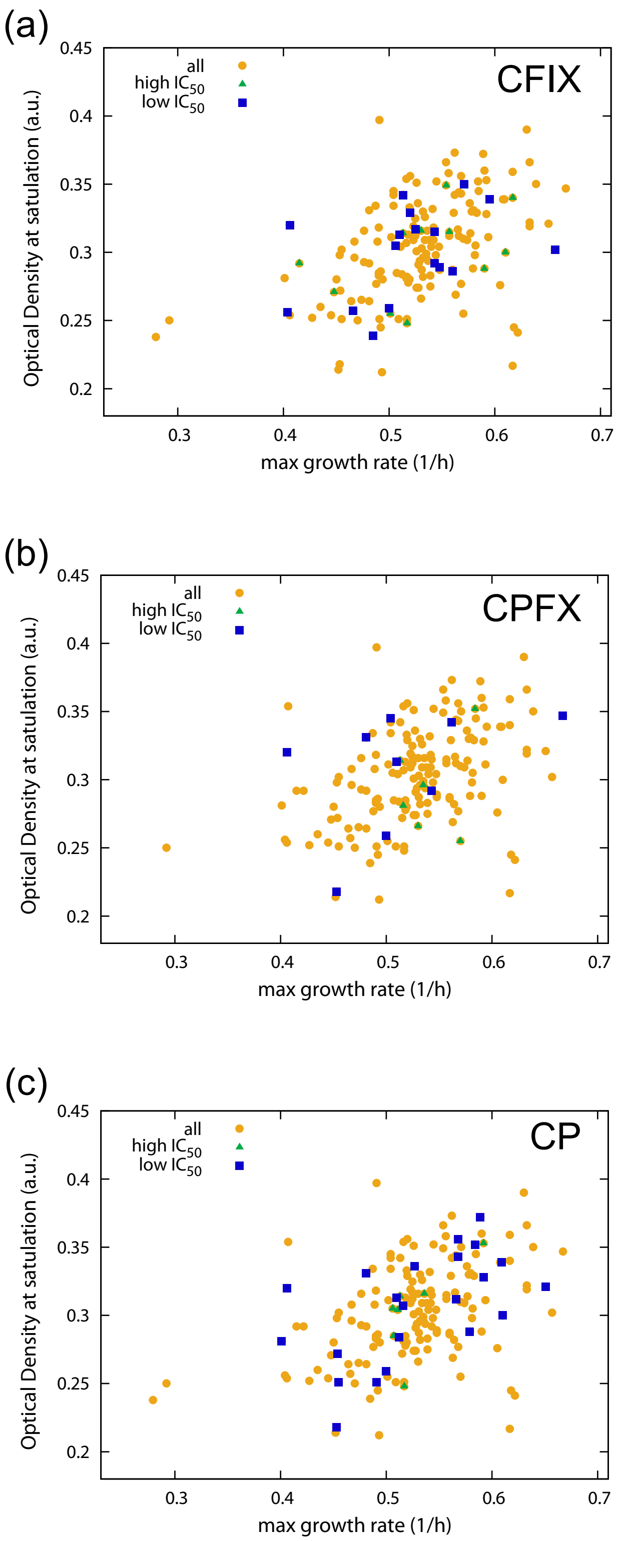

Horinouchi et al, Figure S5

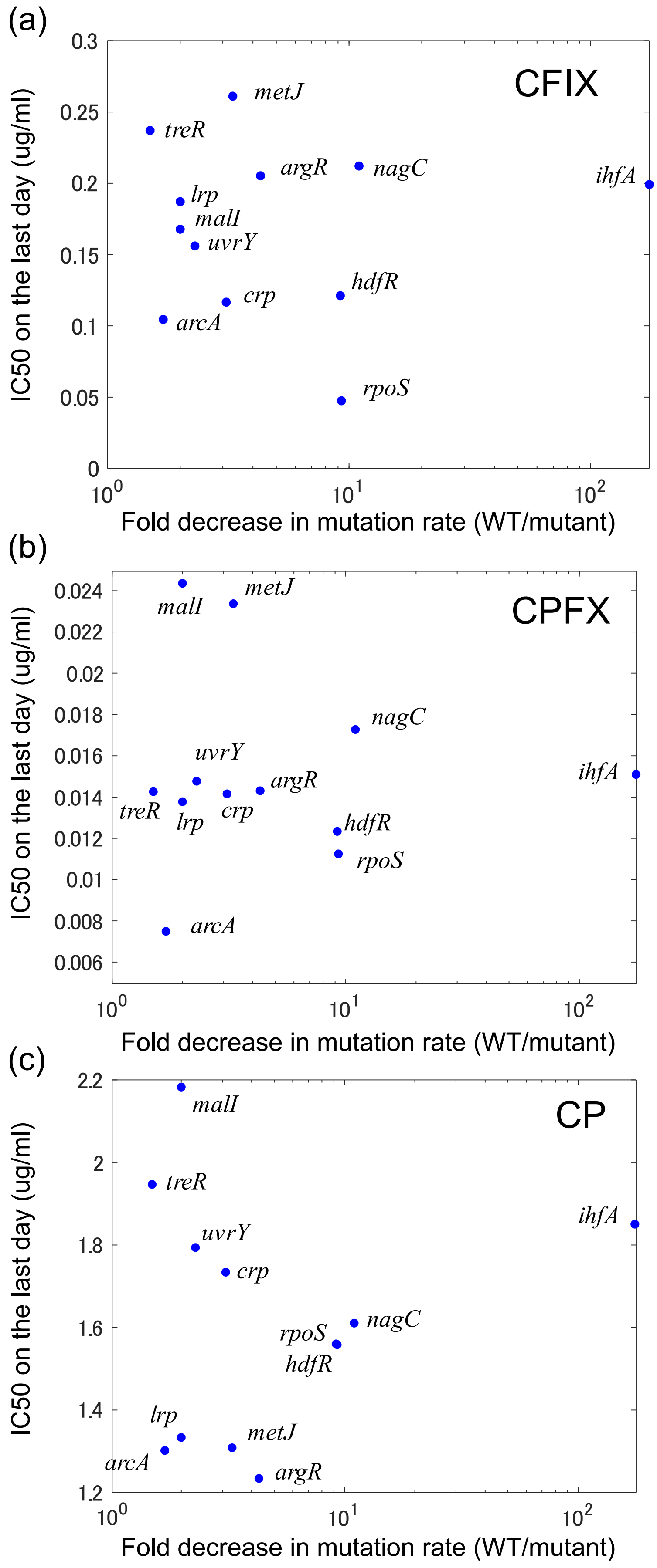

Horinouchi et al, Figure S6

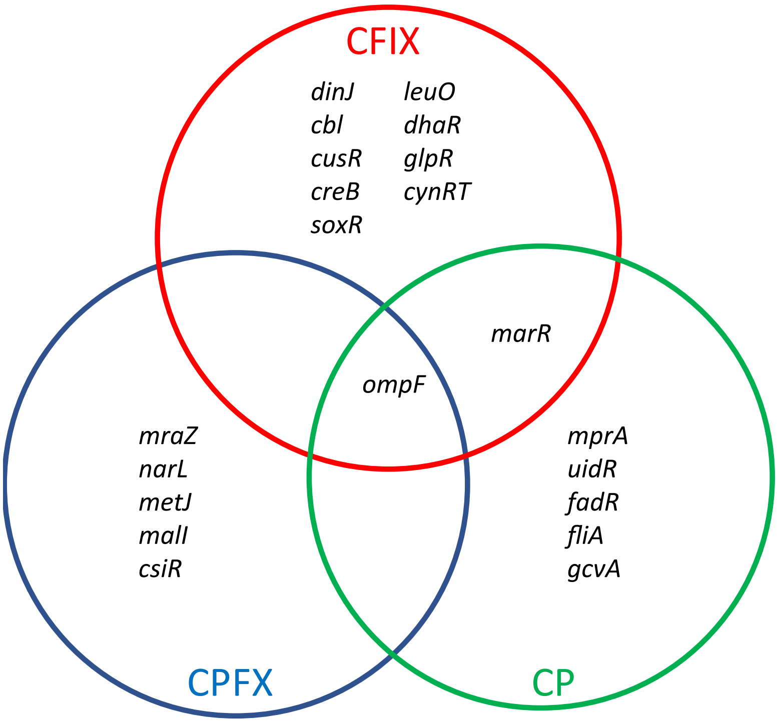

Horinouchi et al, Figure S7

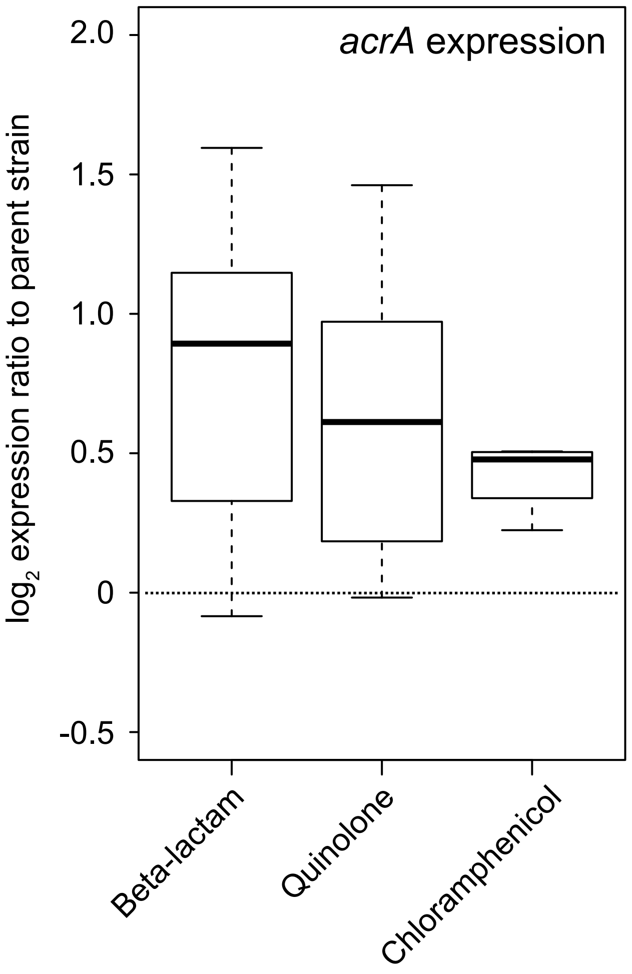

Horinouchi et al, Figure S8

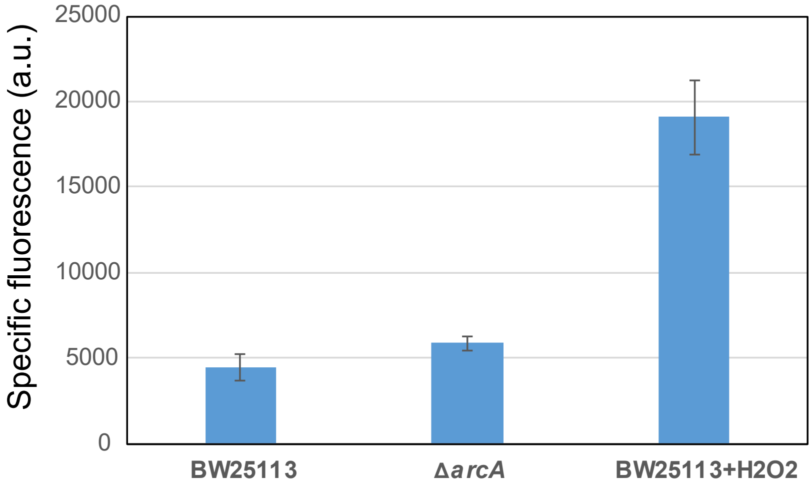

Horinouchi et al, Figure S9
